# Pan-cancer analysis of the angiotensin II receptor-associated protein as a prognostic and immunological gene predicting immunotherapy responses in pan-cancer

**DOI:** 10.3389/fcell.2022.913684

**Published:** 2022-08-19

**Authors:** Kai Hong, Yingjue Zhang, Lingli Yao, Jiabo Zhang, Xianneng Sheng, Lihua Song, Yu Guo, Yangyang Guo

**Affiliations:** ^1^ Department of Thyroid and Breast Surgery, Ningbo City First Hospital, Ningbo, Zhejiang, China; ^2^ Medicine School, Ningbo University, Ningbo, Zhejiang, China; ^3^ Department of Molecular Pathology, Division of Health Sciences, Graduate School of Medicine, Osaka University, Suita, Osaka, Japan

**Keywords:** AGTRAP, pan-cancer, prognosis, tumor microenvironment, immunity

## Abstract

**Background:** Understanding interior molecular mechanisms of tumorigenesis and cancer progression contributes to antitumor treatments. The angiotensin II receptor-associated protein (AGTRAP) has been confirmed to be related with metabolic products in metabolic diseases and can drive the progression of hepatocellular carcinoma and colon carcinoma. However, functions of AGTRAP in other kinds of cancers are unclear, and a pan-cancer analysis of AGTRAP has not been carried out.

**Methods and materials:** We downloaded data from The Cancer Genome Atlas and Genotype-Tissue Expression dataset and The Human Protein Atlas databases and then used R software (version 4.1.1) and several bioinformatic tools to conduct the analysis.

**Results:** In our study, we evaluated the expression of AGTRAP in cancers, such as high expression in breast cancer, lung adenocarcinoma, and glioma and low expression in kidney chromophobe. Furthermore, our study revealed that high expression of AGTRAP is significantly related with poor prognosis in glioma, liver cancer, kidney chromophobe, and so on. We also explored the putative functional mechanisms of AGTRAP across pan-cancer, such as endoplasmic reticulum pathway, endocytosis pathway, and JAK-STAT signaling pathway. In addition, the connection between AGTRAP and tumor microenvironment, tumor mutation burden, and immune-related genes was proven.

**Conclusion:** Our study provided comprehensive evidence of the roles of AGTRAP in different kinds of cancers and supported the relationship of AGTRAP and tumorous immunity.

## Introduction

Cancer is one of the major causes of death in humans. Treatments for various types of cancers have become one of the biggest parts of healthcare spending. Publications reported that abnormal expression and mutation of certain genes including methylation-related genes, immune checkpoint genes, immunomodulatory genes, and mismatch repair (MMR) genes could promote the progression of cancers (Chowell et al., 2021; An and Duan, 2022; Bennett et al., 2022; Kraehenbuehl et al., 2022). In addition, in the past several decades, the tumor microenvironment (TME) has been demonstrated to be a vital factor for cancerous development, such as immune cell infiltration or Hashimoto’s thyroiditis for cancer progression (Zeng et al., 2018; Gan et al., 2021; Mao et al., 2021). Relaying on abundant online databases, many gene-related analyses of cancers were conducted, and a great number of cancer-related genes have been found (Kim et al., 2015; Song et al., 2017; Barneh et al., 2019). Pan-cancer analysis is a valuable way to explore functions and mechanisms of specific genes in various cancers. According to pan-cancer analyses, more detailed research can be designed and conducted.

The renin–angiotensin system (RAS) has been demonstrated to regulate cardiovascular and body fluid homeostasis which causes diseases such as type 2 diabetes mellitus, hypertension, and dyslipidemia (Arendse et al., 2019). Ang II type 1 receptor (AT1R) is an important receptor, and it interacts with the AT1R-associated protein which is encoded by AGTRAP gene, playing a vital role in activating RAS (Daviet et al., 1999; Lopez-Ilasaca et al., 2003). Furthermore, some studies supported that AGTRAP is also associated with tumor progression. Liu et al. (2021) concluded that AGTRAP expression in hepatocellular carcinoma samples is higher than that in normal tissues and adjacent tissues, which potentially leads to a poor prognosis through regulating nuclear factor-kappa B subunit 1 (NF-KB) and mitogen-activated protein kinase (MAPK) signaling pathways. Sanz-Pamplona et al. (2016) reported that overexpression of AGTRAP was found in colon carcinoma which was related to poor outcome in patients. In addition, AGTRAP-BRAF fusion was validated through qRT-PCR and fluorescence *in situ* hybridization (FISH) analysis in gastric cancer (Figueiredo et al., 2017). Still, the underlying functions and mechanisms of AGTRAP are unclear. Further explorations are urgently needed to reveal the precise functions and mechanisms of AGTRAP in cancer patients.

TME is made up of tumor, stromal, endothelial, and immune cells (Downs-Canner et al., 2022). According to previous studies, the component immune cells play an important role in controlling cancer progression (Lam et al., 2021). Hence, more and more researchers tend to apply TME to predict prognosis and drug response. DNA MMR is a pathway of correcting DNA strands which are wrongly synthesized (Mas-Ponte and Supek, 2020). Studies presented that a decrease in MMR activities is related to high microsatellite instability (MSI-H) and high tumor burden (TMB) of tumors (Mandal et al., 2019; Pestana et al., 2022). Simultaneously, in MSI-H tumors, increased tumor-infiltrating lymphocytes (TILs) can be detected (Michael-Robinson et al., 2001). Importantly, researchers have demonstrated how cancers with MMR deficiency benefit from the immune checkpoint blockade can be predicted according to the diversity of MSI (Sahin et al., 2019). In addition, metabolic recombination of tumor cells is a vital method that cancers take to meet their need for growth and their homeostasis in TME (Lunt and Vander Heiden, 2011; Scharping et al., 2021). An and Duan, (2022) suggested that RNA methylation modification plays an important role in metabolic reprogramming which can greatly influence the progression of cancers. These novel markers can be used to predict prognoses of cancer and drug responses and to reveal potential mechanisms of other cancer-related molecules.

In order to explore the functions and mechanisms of AGTRAP in cancers which were highly unknown, we extracted data from online databases to conduct a pan-cancer analysis. Our study analyzed the expression level of AGTRAP and whether it influenced survival, potential mechanisms of AGTRAP, and correlation between AGTRAP and TME, immune cell infiltration, RNA methylation, MMR, immune checkpoint, immunoregulator, TMB, and neoantigen.

## Materials and methods

### Gene and protein expression analysis

First, we analyzed the expression difference of AGTRAP (ENSG00000177674) with the detection data of tumor samples and adjacent normal tissues in The Cancer Genome Atlas (TCGA, https://portal.gdc.cancer.gov/) project from Tumor Immune Estimation Resource (TIMER, http://timer.cistrome.org/). TIMER is a resource of immune infiltration, gene expression profile, clinical prognosis, and somatic mutations and copy number alterations for various kinds of cancer (Li et al., 2017). Inputting “AGTRAP” in the “Gene_DE” module, we found that there are some deficiencies in several cancers [e.g., The Cancer Genome Atlas Adrenocortical Carcinoma (TCGA-ACC) and The Cancer Genome Atlas Uterine Serous Carcinoma (TCGA-USC)]. To further demonstrate the abnormal expression of AGTRAP in diverse cancer types, Gene Expression Profiling Interactive Analysis, version 2 tool (GEPIA2, http://gepia2.cancer-pku.cn/#analysis) was used to conduct the analysis for the combination of TCGA and Genotype-Tissue Expression (GTEx) datasets (Tang et al., 2019). TCGA tumor samples were compared to the normal tissues from TCGA and GTEx [*p*-value cutoff = 0.05, log2 fold change (FC) cutoff = 1]. In order to explore the proteomic expression profile of AGTRAP, we conducted the proteomic expression analysis by UALCAN tool (http://ualcan.path.uab.edu/analysis-prot.html) of the CPTAC (Clinical Proteomic Tumor Analysis Consortium) dataset (Chandrashekar et al., 2017). The keyword “AGTRAP” was input in the “CPTAC analysis” module. The protein expression of AGTRAP in breast cancer, colon cancer, head and neck squamous carcinoma, pancreatic adenocarcinoma, glioblastoma multiforme, and hepatocellular carcinoma was presented, respectively. To discuss AGTRAP expression in different stages of cancer, the “Pathological Stage Plot” module of GEPIA2 was used to visualize the results with violin plots. The log2 [transcripts per million (TPM) +1] was used for log-scale.

### Survival analysis

To explore correlations between AGTRAP expression and different kinds of cancer, the log-rank test and a multivariate Cox proportional hazard regression model were performed. The “overall survival (OS),” “disease-specific survival (DSS),” “disease-free survival (DFS),” and “progression-free survival (PFS)” of various cancers were analyzed using the “survival” R package and visualized by forest plots. The hazard ratios (HRs) and 95% confidence intervals (CIs) calculated through Cox analysis were used to identify the survival difference affected by AGTRAP abnormal expression in various types of cancer. The median expression of AGTRAP was used to divide samples into low- and high-expression groups.

### Protein–protein interaction network

A protein–protein interaction (PPI) network for AGTRAP was constructed using the STRING website tool (https://string-db.org/) (Szklarczyk et al., 2019). The medium confidence genes were conserved (interaction score ≥0.4). Then, correlation analysis of AGTRAP and its related genes in pan-cancer was visualized using the GEPIA2 tool (Tang et al., 2019). We used Pearson’s test for the correlation analysis, nonlog scale for calculation, and log-scale axis for visualization. Moreover, the expression of AGTRAP-related genes across pan-caner was visualized by the heatmap using the GEPIA2 tool, and log2 (TPM +1) was used for log-scale (Tang et al., 2019). In addition, OS and DFS of AGTRAP-related genes were also analyzed using the GEPIA2 tool (Tang et al., 2019).

### Enrichment analysis

Gene Ontology (GO) and Kyoto Encyclopedia of Genes and Genomes (KEGG) enrichment analyses were conducted to analyze the potential functions of AGTRAP (Kanehisa and Goto, 2000; The Gene Ontology Consortium, 2019). First, we selected the most relevant top 200 genes of AGTRAP through the GEPIA2 tool (Supplementary Material S1). Then, functional annotations and enrichment pathways of AGTRAP and its relevant genes were identified. Moreover, gene set enrichment analysis (GSEA) of single AGTRAP was separately performed in different kinds of cancers (Subramanian et al., 2005). Enrichment analysis was conducted using “limma,” “org.Hs.eg.db,” “clusterprofiler,” and “enrichplot” R packages (Yu et al., 2012).

### Immune score and cell infiltration analysis

We downloaded the standardized pan-cancer dataset from the University of California, Santa Cruz (UCSC, https://xenabrowser.net/) database including TCGA, Therapeutically Applicable Research to Generate Effective Treatments (TARGET), and GTEx cohorts and then extracted the expression data of AGTRAP gene in each sample (Kent et al., 2002). The results of AGTRAP expression were transformed to log(x+0.001), and the gene expression profiles of each kind of tumors were extracted. Afterward, we evaluated the correlation between AGTRAP and immune scores (ImmuneScore, StromaScore, and MicroenvironmentScore) and immune cell infiltration in different kinds of cancers by xCell algorithms using the “IOBR” R package (Zeng et al., 2021). Finally, the significance was identified using Pearson’s correlation coefficient by the “psych” R package from the 10,180 samples of 44 types of cancers (Revelle and Revelle, 2015).

### Immune checkpoint, RNA modification, and immunoregulator correlation analysis

In order to evaluate the correlation between immune checkpoint, RNA modification, and immunoregulation genes and AGTRAP, Spearman correlation analysis was conducted to analyze the data downloaded from the UCSC database (Myers and Sirois, 2004). Regarding the immune checkpoint, inhibitory and stimulatory genes were analyzed, respectively. In addition, the correlation between “N6-methyladenosine (m(6)A),” “N1-methyladenosine (m(1)A),” and “5-methylcytosine (m(5)C)” modifications and AGTRAP was presented, respectively. Moreover, the relationship between immunoregulation genes and AGTRAP was estimated.

### Tumor mutational burden, microsatellite instability, neoantigen, and mismatch repair mutation correlation analysis

The mutation data of each kind of cancer were calculated using the “MAF Tools” R package (Mayakonda et al., 2018). MSI scores, neoantigen data, and MMR data were obtained from previous studies. MMR genes included: epithelial cell adhesion molecule (EPCAM), PMS1 homolog 2 (PMS2), MutS homolog 6 (MSH6), MutS protein homolog 2 (MSH2), and MutL protein homolog 1 (MLH1) (Latham et al., 2019). Finally, cancers with a number less than 3 were removed from the results. The correlation was analyzed between TMB, MSI scores, neoantigen, and MMR and AGTRAP expression by Spearman’s method (Revelle and Revelle, 2015).

### Immunohistochemistry

The immunohistochemistry results of AGTRAP pathological expression in diverse cancers were obtained from The Human Protein Atlas database (THPA, https://www.proteinatlas.org/) (Thul and Lindskog, 2018).

### Cell culture and transfection

The breast, pancreatic, and gastric cancer cells (BT-549, PANC-1, and HGC-27) and normal cells (MCF-10A, HPNE, and GES-1) were obtained from the Cell Bank of the Shanghai Institute of Biochemistry and Cell Biology (Shanghai, China). The cell lines were cultivated in Dulbecco’s modified Eagle’s medium or RPMI-1640, both added with 10% fetal bovine serum (FBS), 100 μg/ml streptomycin, and 100 U/ml penicillin (Gibco). The cells were infected with si-AGTRAP (GenePharma).

### qRT-PCR

All cells were washed with PBS and then homogenized in TRIzol reagent (Invitrogen). Total RNA was extracted and reverse transcribed into the cDNA template, and then SYBR Green Real-Time PCR Master Mix Plus (Toyobo) was used for qRT-PCR. β-Actin was used as an endogenous reference gene. The primer sequences for amplification were as follows: forward 5′-TGG​GGC​TGC​ATT​GTA​TTC​TCA-3′ and reverse 5′-AGC​CAC​CCA​GAA​ACA​TGC​TTA-3′ for AGTRAP and forward 5′-TGA​CGT​GGA​CAT​CCG​CAA​AG-3′ and reverse 5′-CTG​GAA​GGT​GGA​CAG​CGA​GG-3′ for β-actin.

### Western blot

To measure the protein expression of AGTRAP and its related signaling pathway, Western blot was conducted on breast cancer, pancreatic cancer, and gastric cancer. Cell proteins were extracted in accordance with manufacturer’s instruction. The concentration of proteins was identified using BCA protein assay kit (Beyotime, Shanghai, China). A volume of 35 µL of proteins from each sample was used for electrophoresis. Membranes were incubated overnight with primary antibodies followed by blocking the membranes with 5% skimmed milk. Subsequently, secondary antibodies (1:5,000) were used for further incubation. Finally, the protein bands were visualized through an enhanced chemiluminescent reagent (Thermo Fisher).

The AGTRAP antibody (F-6) (sc-271367) was purchased from Santa Cruz Biotechnology (Heidelberg, Germany). The mTOR antibody (ab2732) and AKT antibody (ab8805) were purchased from Abcam (Beijing, China). The p-mTOR antibody (CST 5536S) and p-AKT antibody (CST 4060S) were purchased from Cell Signaling Technology (CST, United States). The GAPDH antibody (AP0063) was purchased from Bioworl Technology (United States).

### Statistical analysis

The Wilcoxon test was used to compare the data of tumor samples and normal tissues. K–M analysis and Cox regression analysis were used to analyze the survivals. In addition, Spearman’s test or Pearson’s test was used to conduct the correlation analyses. *p*-values less than 0.05 were considered statistically significant. Annotations were used to show statistical significance: *p < 0.05, **p < 0.01, ***p < 0.001, and ****p < 0.0001. R software (version 4.1.1), GraphPad Prism (version 8.4.3), and several bioinformatical tools such as Sangerbox (http://vip.sangerbox.com/home.html) and TIMER were used to conduct the statistical analyses.

## Results

### Angiotensin II receptor-associated protein expression in pan-cancer

**FIGURE 1:**
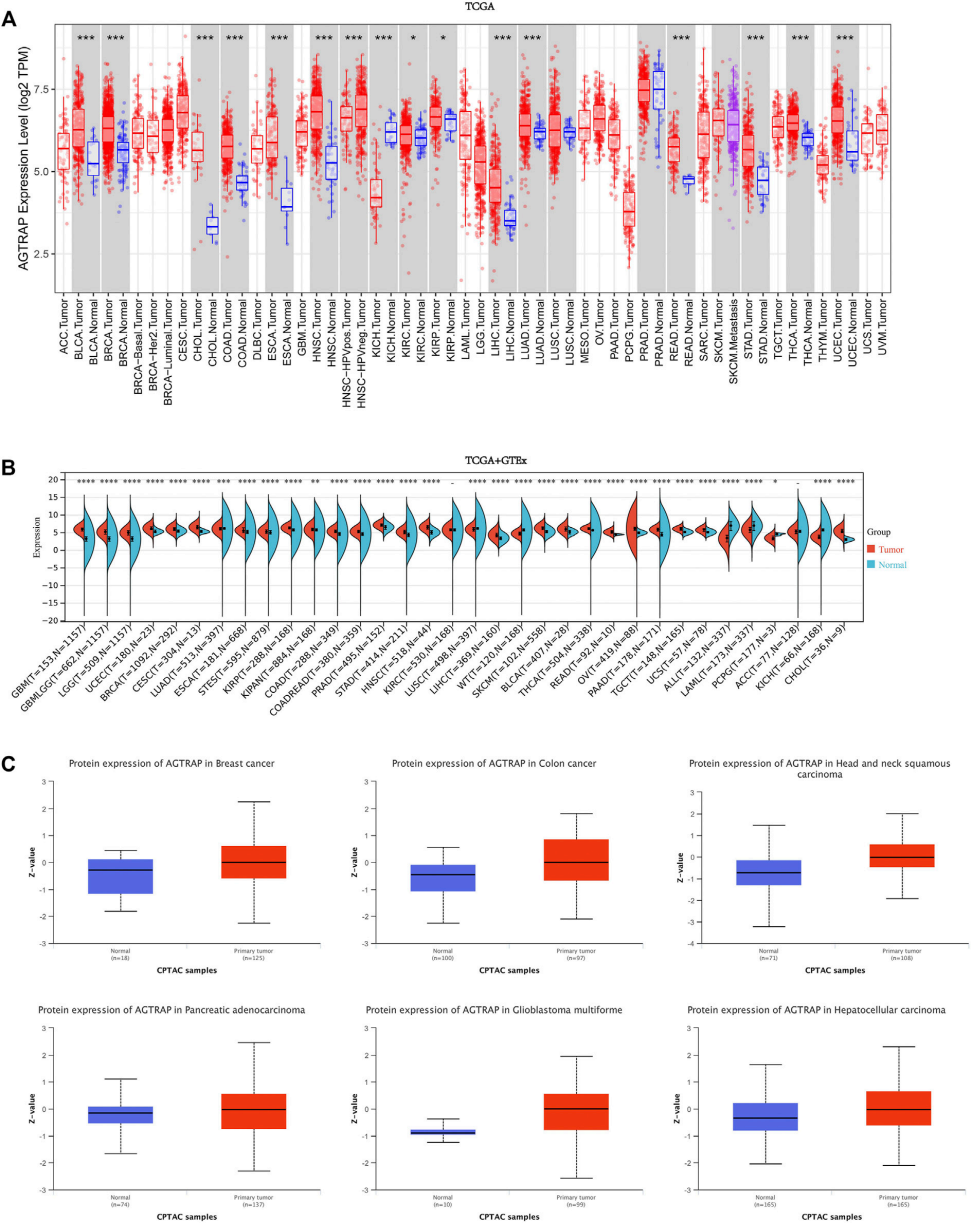
Expression and protein level of AGTRAP in pan-cancer. **(A)** mRNA expression levels of AGTRAP in different cancers from TCGA data in TIMER. *p < 0.05, **p < 0.01, ***p < 0.001. **(B)** Analysis of TCGA and GTEx datasets showed differential mRNA expression of AGTRAP in various cancers. *p < 0.05, **p < 0.01, ***p < 0.001. **(C)** AGTRAP protein expression level in pan-cancer from CPTAC samples. Z-values represented standard deviations from the median across samples for the given cancer types.

First, AGTRAP mRNA expression in tumor samples and normal tissues was analyzed across TCGA dataset (Figure 1A). AGTRAP expression in tumor samples of BLCA (bladder urothelial carcinoma), BRCA (breast invasive carcinoma), CHOL (cholangiocarcinoma), COAD (colon adenocarcinoma), ESCA (esophageal carcinoma), HNSC (head and neck squamous cell carcinoma), LIHC (liver hepatocellular carcinoma), LUAD (lung adenocarcinoma), READ (rectum adenocarcinoma), STAD (stomach adenocarcinoma), THCA (thyroid carcinoma), UCEC (uterine corpus endometrial carcinoma) (*p* < 0.001), KIRC (kidney renal clear cell carcinoma), and KIRP (kidney renal papillary cell carcinoma) (*p* < 0.05) is significantly higher than that in normal tissues. Moreover, in KICH (kidney chromophobe), AGTRAP expression in tumor samples is significantly lower than that in normal tissues (*p* < 0.001). Notably, HPV-negative HNSC has a higher AGTRAP expression than HPV-positive HNSC.

In view of the deficiency of several kinds of normal tissue data, TCGA and GTEx datasets were pooled together for analyzing. As shown in Figure 1B, significantly higher AGTRAP expression in tumor samples than in normal tissues was presented in GBM (glioblastoma multiforme), LGG (brain lower grade glioma), UCEC, BRCA, CESC (cervical squamous cell carcinoma and endo-cervical adenocarcinoma), ESCA, STES (stomach and esophageal carcinoma), KIRP, COAD, PRAD (prostate adenocarcinoma), STAD, HNSC, LIHC, SKCM (skin cutaneous melanoma), BLCA, THCA, READ, OV (ovarian serous cystadenocarcinoma), PAAD (pancreatic adenocarcinoma), TGCT (testicular germ cell tumors), UCS (uterine carcinosarcoma), CHOL (*p* < 0.0001), LUAD (*p* < 0.001), and KIPAN (pan-kidney) (*p* < 0.01). Significantly lower AGTRAP expression in tumor samples than in normal tissues was demonstrated in LUSC (lung squamous cell carcinoma), ALL (acute lymphoblastic leukemia), LAML (acute myeloid leukemia), KICH (*p* < 0.0001), and PCPG (pheochromocytoma and paraganglioma) (*p* < 0.05).

Abnormal levels of cancer-related proteins are strongly related to cancer progression. Thus, in addition to the mRNA expression analysis, AGTRAP data from CPTAC was analyzed by comparing the tumor samples to normal tissues. Finally, significant high expression of AGTRAP was found in breast cancer, colon cancer, head and neck squamous carcinoma, pancreatic adenocarcinoma, glioblastoma multiforme, and hepatocellular carcinoma (Figure 1C).

**FIGURE 2:**
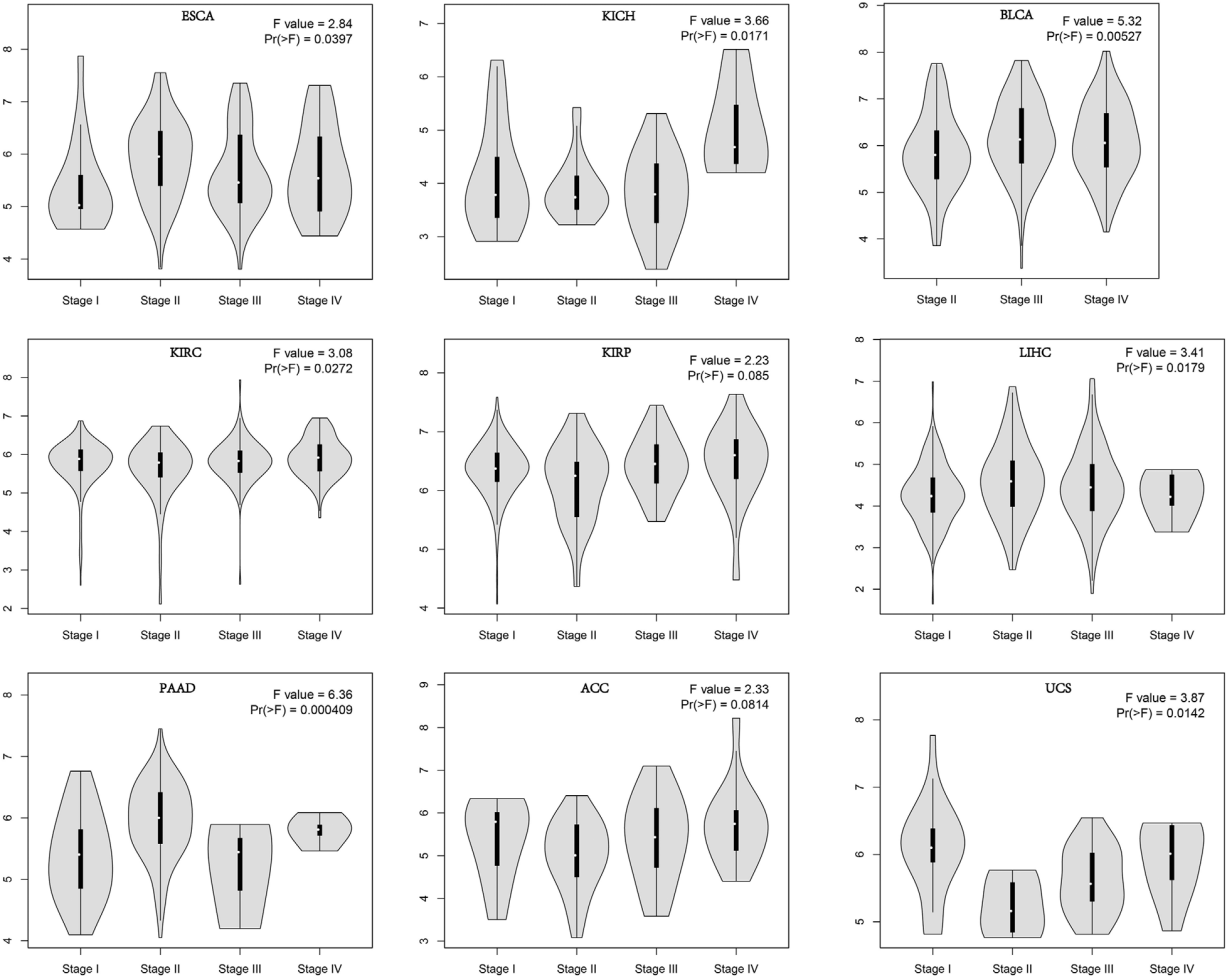
Stage-dependent expression level of AGTRAP. Stages (stage I, stage II, stage III, and stage IV) of pan-cancer were analyzed using TCGA dataset. The log2 (TPM+1) was used for log-scale.

In order to evaluate the expression of AGTRAP in different pathological stages in pan-cancer, we conducted the “Pathological Stage Plot” module in GEPIA2. A significant difference of AGTRAP expression in different pathological stages was demonstrated across various cancer types, such as ESCA, KICH, BLCA, KIRC, LIHC, PAAD, and UCS (*p* < 0.05) (Figure 2).

### Survival analysis of abnormal angiotensin II receptor-associated protein expression in pan-cancer

**FIGURE 3:**
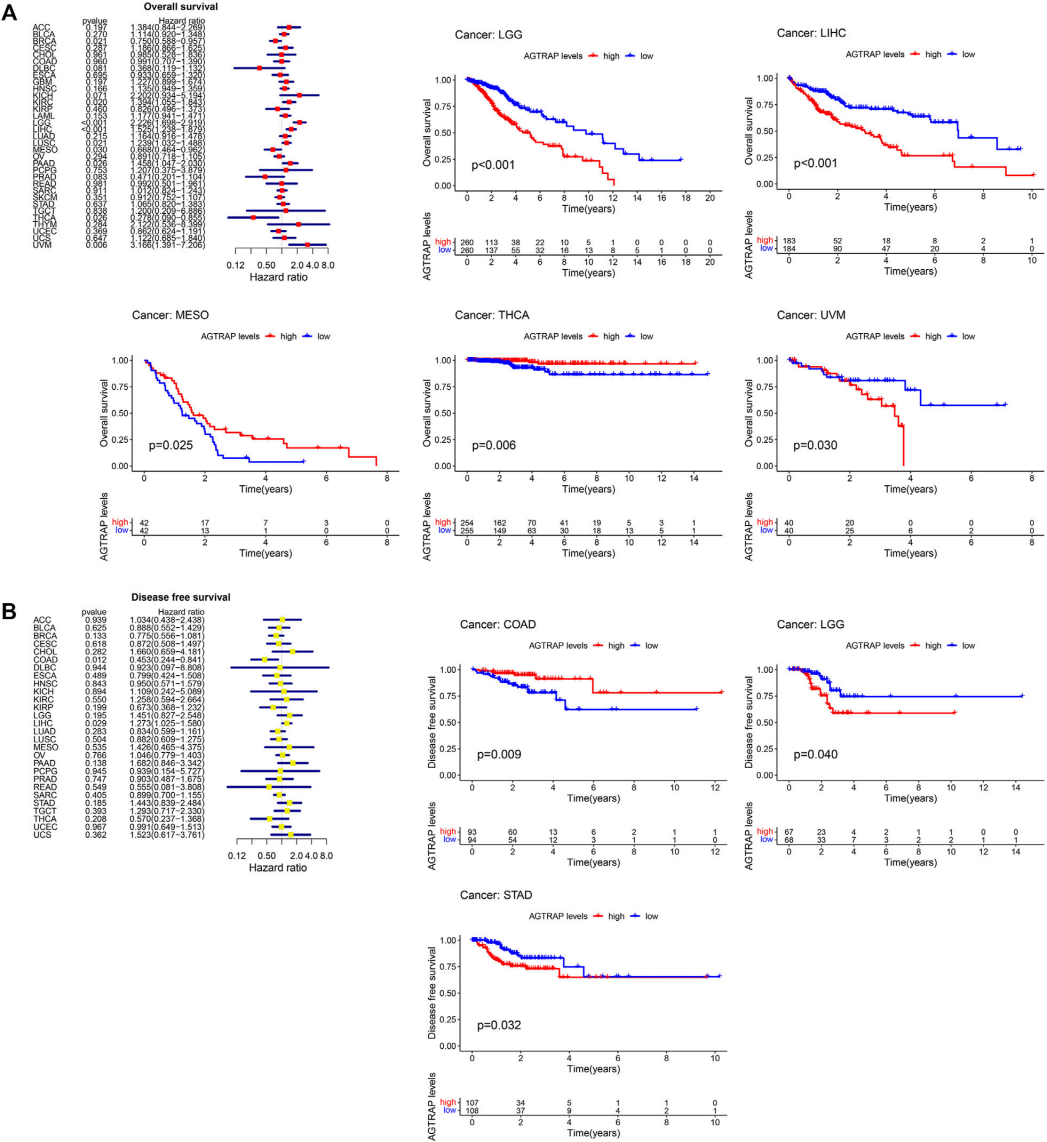
Correlation between the expression of AGTRAP and prognosis in pan-caner. **(A)** Relationship of overall survival (OS) and AGTRAP expression in pan-cancer. **(B)** Relationship of disease-free survival (DFS) and AGTRAP expression in pan-cancer.

**FIGURE 4:**
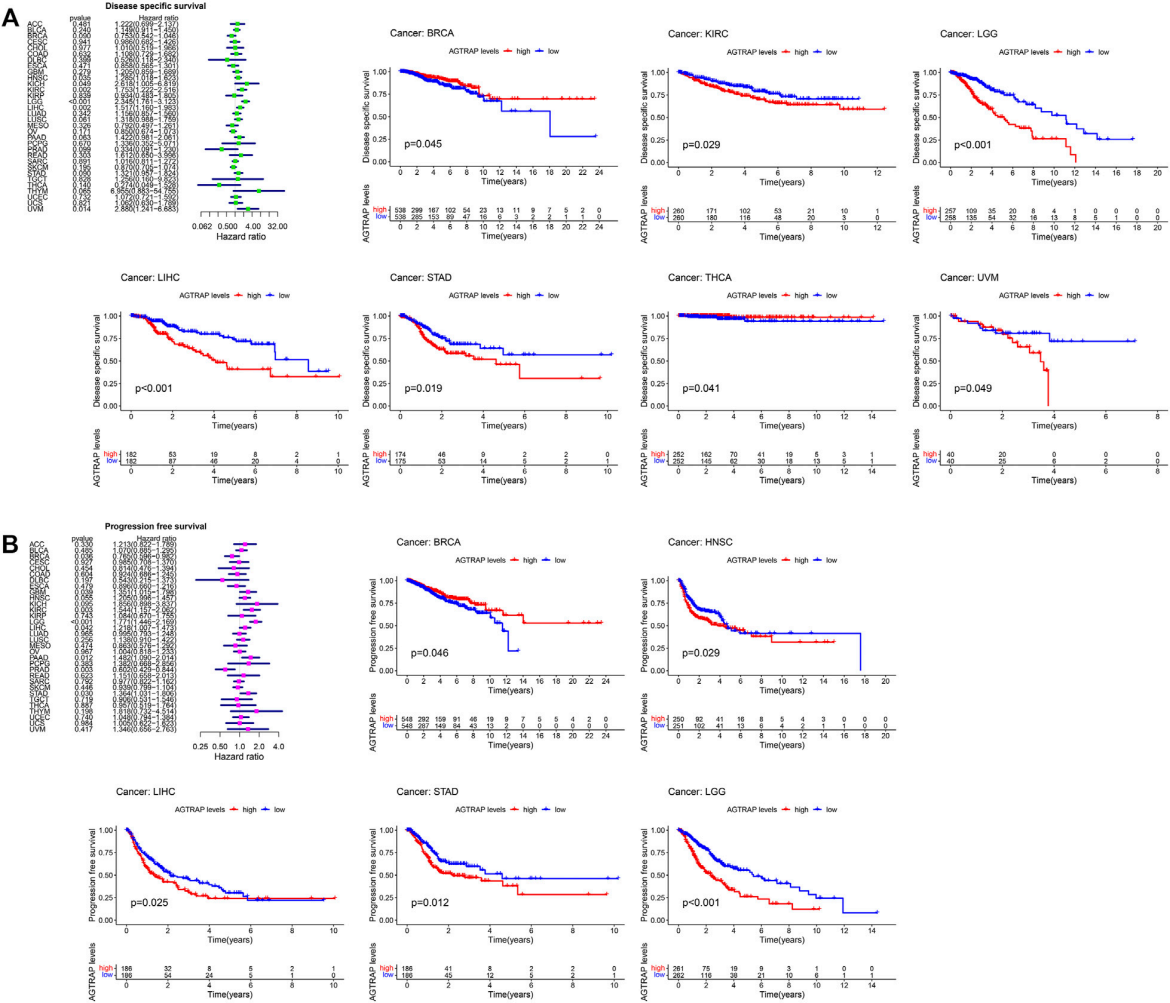
Correlation between the expression of AGTRAP and prognosis in pan-caner. **(A)** Relationship of disease-specific survival (DSS) and AGTRAP expression in pan-cancer. **(B)** Relationship of progression-free survival (PFS) AGTRAP expression in pan-cancer.

TCGA cancer cases were divided into low- and high-expression subsets according to AGTRAP expression, and then the correlation between AGTRAP expression and prognosis was analyzed. Highly expressed AGTRAP was associated to significantly poor OS in LGG, LIHC, and UVM (uveal melanoma) (*p* < 0.05) (Figure 3A), significantly poor DFS in LGG and STAD (*p* < 0.05) (Figure 3B), significantly poor DSS in KIRC, LGG, LIHC, STAD, and UVM (Figure 4A), and significantly poor PFS in LGG, HNSC, LIHC, and STAD (*p* < 0.05) (Figure 4B). Contrarily, highly expressed AGTRAP showed better OS in MESO (mesothelioma) and THCA (*p* < 0.05) (Figure 3A), better DFS in COAD (*p* < 0.05) (Figure 3B), better DSS in BRCA and THCA (*p* < 0.05) (Figure 4A), and better PFS in BRCA (*p* < 0.05) (Figure 4B).

### Protein–protein interaction network and correlation and survival analyses of angiotensin II receptor-associated protein-related genes

**FIGURE 5:**
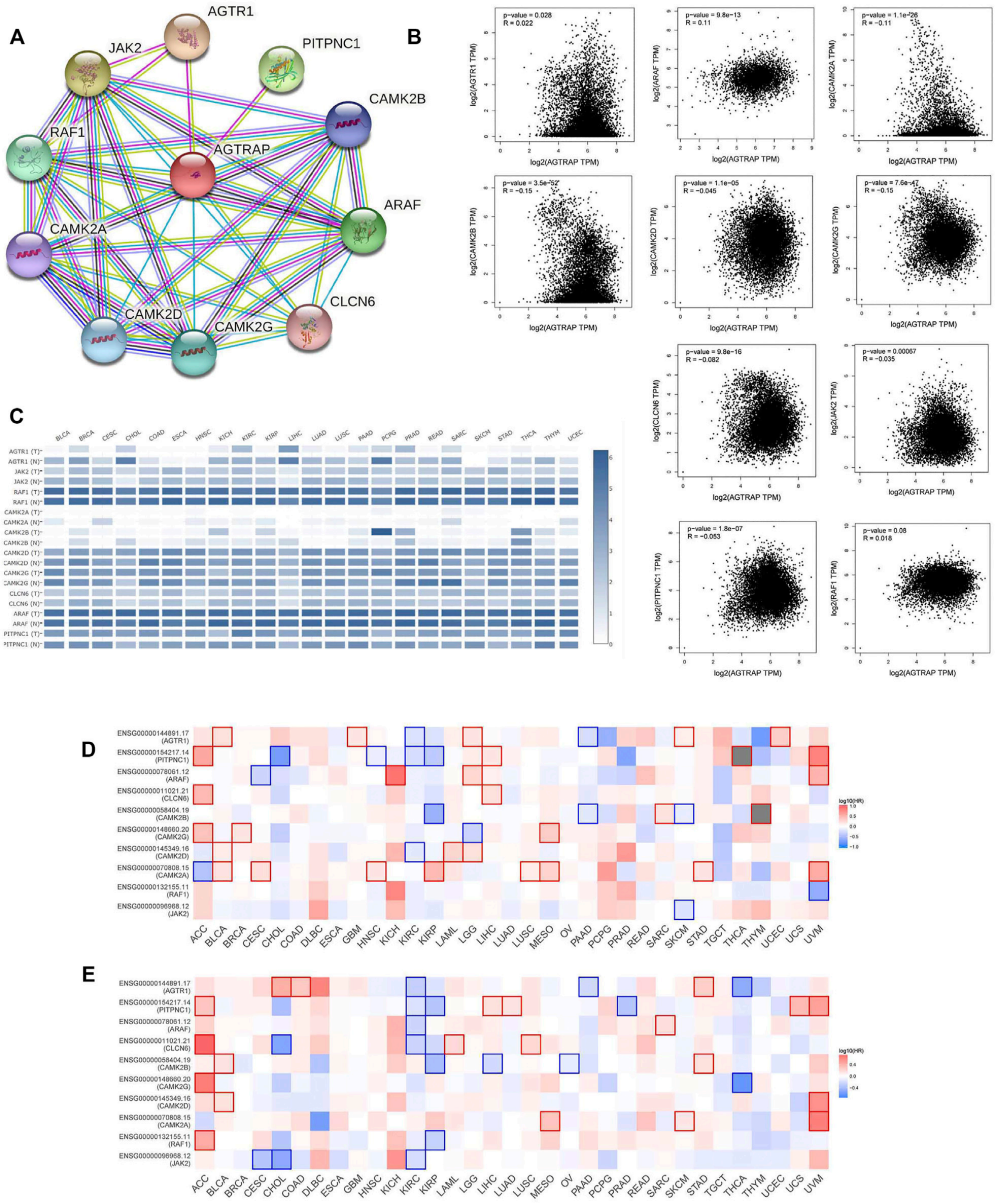
Analysis of top 10 AGTRAP-related genes. **(A)** AGTRAP interaction network. **(B)** Expression correlation between AGTRAP and AGTRAP-related genes (AGTR1, JAK2, RAF1, CAMK2A, CAMK2B, CAMK2D, CAMK2G, CLCN6, ARAF, and PITPNC1). **(C)** Expression of AGTRAP-related genes in different cancer samples and normal tissues. **(D)** Overall survival (OS) analysis of AGTRAP-related genes in pan-cancer. **(E)** Disease-free survival (DFS) analysis of AGTRAP-related genes in pan-cancer.

**FIGURE 6:**
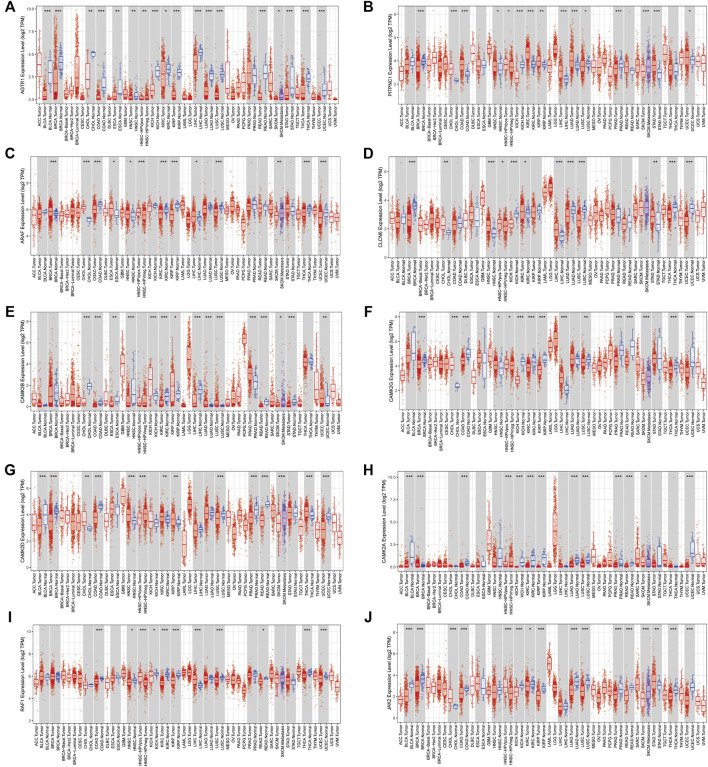
Expression of AGTRAP-related genes in pan-cancer. **(A)** AGTR1. **(B)** PITPNC1. **(C)** ARAF. **(D)** CLCN6. **(E)** CAMK2B. **(F)** CAMK2G. **(G)** CAMK2D. **(H)** CAMK2A. **(I)** RAF1. **(J)** JAK2.

The top 10 related genes of AGTRAP were identified through STRING, namely, AGTR1, JAK2, RAF1, CAMK2A, CAMK2B, CAMK2D, CAMK2G, CLCN6, ARAF, and PITPNC1 (Figure 5A). As shown in Figure 5B, the expression of AGTRAP was positively correlated with AGTR1 and ARAF. A significantly negative correlation was confirmed between AGTRAP and CAMK2A, CAMK2B, CAMK2D, CAMK2G, CLCN6, JAK2, and PITPNC1. In addition, difference in the expression of AGTRAP-related genes was found in various cancers in TCGA pan-cancer datasets (Figure 5C). A significant difference in OS and RFS of the AGTRAP-related genes was presented in the heatmaps. Notably, the same gene shows contrary affects in different cancers. Survival analysis showed that the high expression of AGTRAP-related genes mostly leads to poor prognoses in ACC, BLCA, KICH, LGG, LIHC, LUSC, MESO, SARC (sarcoma), and UVM and leads to better outcomes in CHOL, KIRC, KIRP, and PAAD (Figures 5D,E). In addition, to better represent the expression of AGTRAP-related genes in pan-cancer, we conducted the analysis in the TIMER database. The results showed that the AGTRAP-related genes have significantly abnormal expression in diverse cancer types. (Figure 6).

### Angiotensin II receptor-associated protein and related gene functional enrichment analysis

**FIGURE 7:**
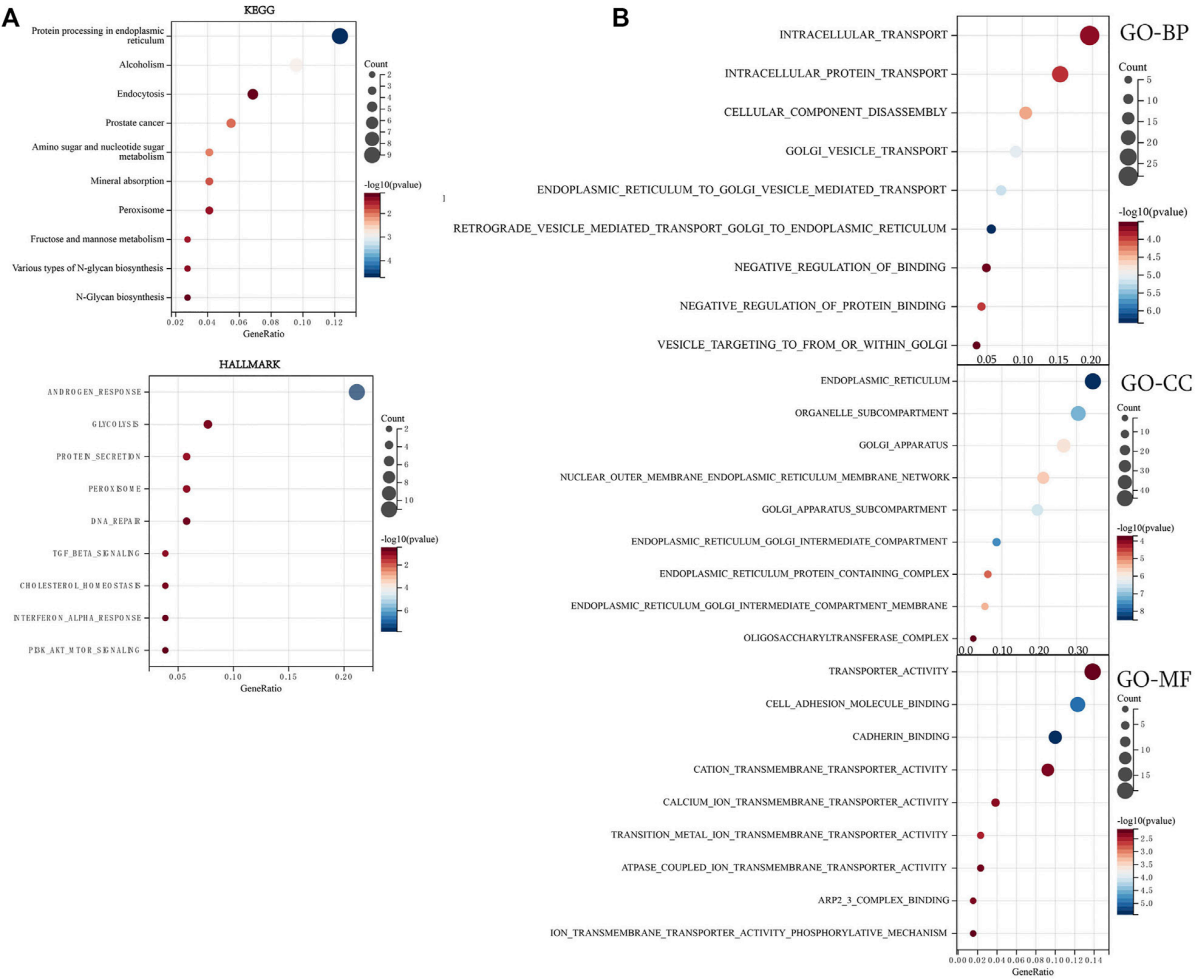
Enrichment analysis of top 200 AGTRAP-related genes. **(A)** KEGG enrichment analysis of AGTRAP-related genes. **(B)** GO enrichment analysis of AGTRAP-related genes.

To further explore the molecular mechanisms of AGTRAP in pan-cancer, enrichment analysis was conducted using the data of AGTRAP and its top 200 related genes. As shown in Figure 7A, AGTRAP is mostly involved in “protein processing in the endoplasmic reticulum pathway,” “alcoholism pathway,” “endocytosis pathway,” “prostate cancer pathway,” and “amino sugar nucleotide sugar metabolism pathway.” In addition, AGTRAP is mostly enriched in hallmarks such as “androgen response,” “glycolysis,” “protein secretion,” “peroxisome,” and “DNA repair.” Regarding the GO enrichment analysis, “intracellular transport,” “intracellular protein transport,” “cellular component disassembly,” and “golgi vesicle transport” are the most relative functional annotations of biological process; “endoplasmic reticulum,” “organelle subcompartment,” “golgi apparatus,” and “nuclear outer membrane endoplasmic reticulum membrane network” are mostly related to cellular component; “transporter activity,” “cell adhesion molecule binding,” “cadherin binding,” and “cation transmembrane transport activity” are significantly associated to molecular function (Figure 7B).

**FIGURE 8:**
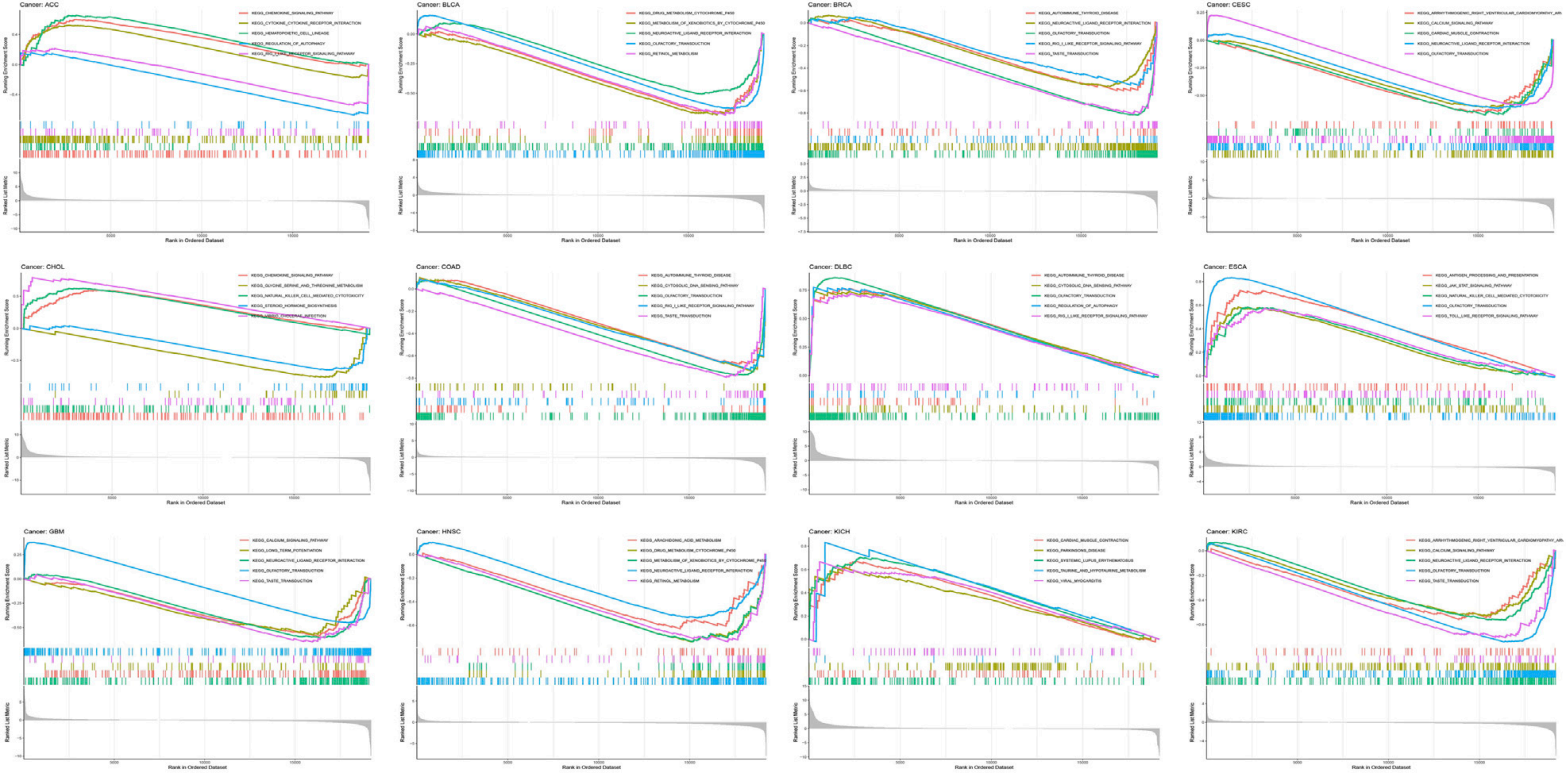
GSEA of AGTRAP in pan-cancer.

Afterward, enrichment pathways of single AGTRAP in different kinds of cancers were evaluated to further reveal the potential mechanism (Figure 8). From GSEA, enrichment of “olfactory transduction pathway” was found in most types of cancers including DLBC, ESCA, BLCA, BRCA, CESC, COAD, GBM, and KIRC. In addition, the “neuroactive ligand–receptor interaction pathway” was enriched in BLCA, BRCA, CHOL, GBM, HNSC, and KIRC. Notably, the “autoimmune thyroid disease pathway,” “cytosolic DNA sensing pathway,” “olfactory transduction pathway,” “RIG-I-like receptor signaling pathway,” “neuroactive ligand–receptor interaction pathway,” and “taste transduction pathway” were enriched in DLBC, BRCA, and COAD, which means that AGTRAP might have some similar functions in these three kinds of cancers. Importantly, AGTRAP also enriched in some immune-related pathways including “chemokine signaling pathway,” “cytokine–receptor interaction pathway,” “natural killer cell-mediated cytotoxicity pathway,” “autoimmune thyroid disease pathway,” “cytosolic DNA sensing pathway,” “regulation of autophagy pathway,” “RIG-I-like receptor signaling pathway,” “antigen processing and presentation pathway,” “JAK-STAT signaling pathway,” “toll-like receptor signaling pathway,” and “neuroactive ligand–receptor interaction pathway” and some metabolism-related pathways including “taurine and hypotaurine metabolism pathway,” “drug metabolism cytochrome P450 pathway,” “metabolism of xenobiotics by the cytochrome P450 pathway,” “retinol metabolism pathway,” “glycine, serine, and threonine metabolism pathway,” and “arachidonic acid metabolism pathway,” which confirmed that AGTRAP is a gene whose functions are associated with immunity and metabolism.

### Tumor microenvironment correlation analysis

**FIGURE 9:**
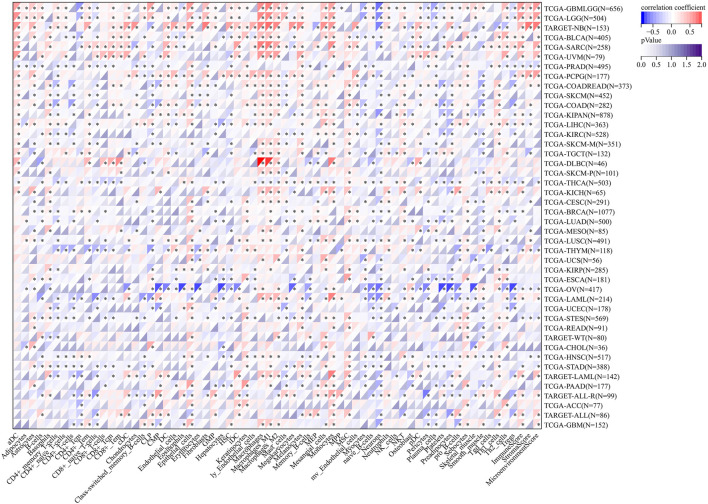
Analysis of correlation between the expression of AGTRAP and immune cell infiltration, ImmuneScore, StromaScore, and MicroenvironmentScore.

As shown in Figure 9, a significantly positive association was found between AGTRAP and ImmuneScore in LGG, NB (neuroblastoma), BLCA, SARC, UVM, PCPG, KIPAN, LIHC, KICH, LAML, ALL, and GBM; between AGTRAP and StromalScore in LGG, NB, PCPG, COAD, LUSC, and THYM; and between AGTRAP and MicroenvironmentScore in LGG, NB, BLCA, SARC, UVM, PCPG, LAML, ALL, and GBM. Moreover, a significantly negative correlation was confirmed between AGTRAP and ImmuneScore in THCA, BRCA, THYM, OV, STES, and STAD; between AGTRAP and StromalScore in PRAD, KIPAN, LIHC, TGCT, CESC, MESO, OV, STES, and HNSC; and between AGTRAP and MicroenvironmentScore in PRAD, THCA, THYM, OV, STES, HNSC, and STAD.

Then, immune cell infiltration analysis was conducted to further expose the association between AGTRAP expression and tumor microenvironment in pan-cancer (Figure 9). The heatmap shows that in almost all kinds of cancers, AGTRAP expression is variously related with immune and stromal cells. Notably, LGG, NB, BLCA, SARC, UVM, and PCPG are cancers which have more positive coexpression of AGTRAP and cell infiltration, while OV, THYM, LAML, UCEC, and STES have more negative coexpression. In addition, M2 macrophages, mesangial cells, monocytes, and epithelial cells show positive coexpression in most kinds of cancers and CD4^+^ T cells including memory and naïve cells, myocytes, naïve B cells, neutrophils, pericytes, plasma cells, and platelets show negative coexpression in most cancers.

### Immune checkpoint, RNA methylation, and immunomodulator correlation analysis

**FIGURE 10:**
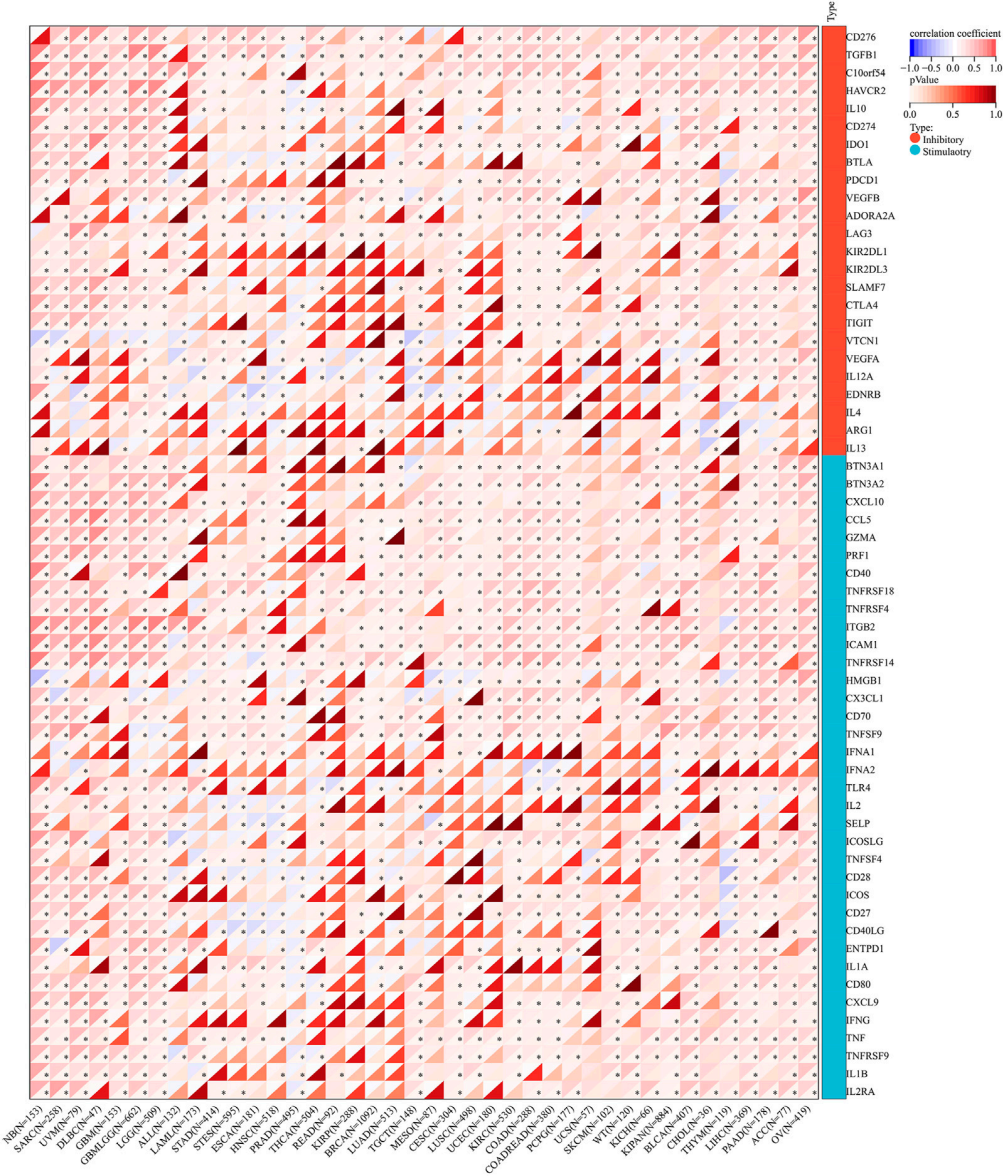
Analysis of correlation between the expression of AGTRAP and immune checkpoint genes.

The correlation of AGTRAP expression and 60 immune checkpoint genes including inhibitory and stimulatory genes was analyzed (Figure 10). AGTRAP expression in different kinds of cancer is variously associated with immune checkpoint genes. In NB, SARC, UVM, DLBC, GBM, LGG, TGCT, KIRC, COAD, PCPG, SKCM, KIPAN, BLCA, CHOL, THYM, LIHC, PAAD, ACC, and OV, AGTRAP expression was more strongly related with immune checkpoint genes than others. In addition, CD276, TGFB1, C10orf54, IL10, CD274, BTN3A1, BTN3A2, CXCL10, CCL5, GZMA, PRF1, CD40, TNFRSF18, TNFRSF4, TNFRSF14, TNFRSF9, ITGB2, ICAM1, HMGB1, CX3CL1, CD70, ICOS, CD27, CD40LG, ENTPD1, IL1A, CD80, CXCL9, IFNG, TNF, IL1B, and IL2RA were more significantly associated with AGTRAP. From the result, it can obviously be concluded that AGTRAP is more related with the stimulatory immune checkpoint.

**FIGURE 11:**
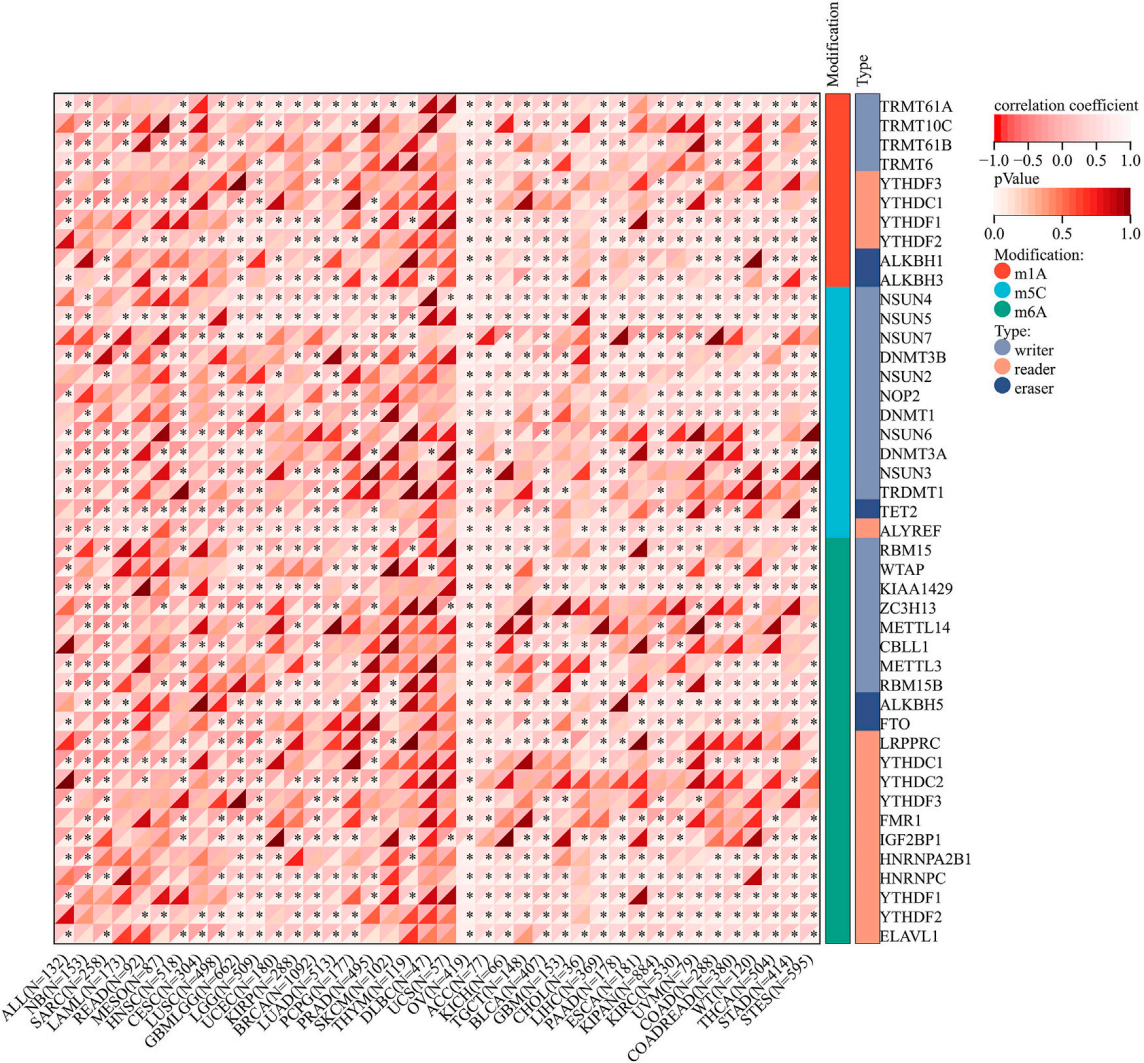
Analysis of correlation between the expression of AGTRAP and RNA modification genes.

Regarding the RNA modification genes, correlation analysis was performed for these genes and AGTRAP across various cancers. As shown in Figure 11, AGTRAP expression in ALL, NB, SARC, HNSC, LUSC, LGG, OV, ACC, KICH, TGCT, BLCA, GBM, CHOL, LIHC, PAAD, ESCA, KIPAN, KIRC, UVM, COAD, WT (Wilms’ tumor), THCA, STAD, and STES relates with more modification genes. Moreover, higher correlation modification genes include: m(1)A writer: TRMT61A; m(1)A readers: TYHDF1 and TYHDF2; m(1)A eraser: ALKBH3; m(5)C writers: NSUN4 and NSUN5; m(5)C reader: ALYREF; m(5)C eraser: TET2; m(6)A writers: KIAA1429; m(6)A readers HNRNPA2B1, HNRNPC, YTHDF1, YTHDF2, and ELAVL1; and m(6)A eraser: ALKBH5.

**FIGURE 12:**
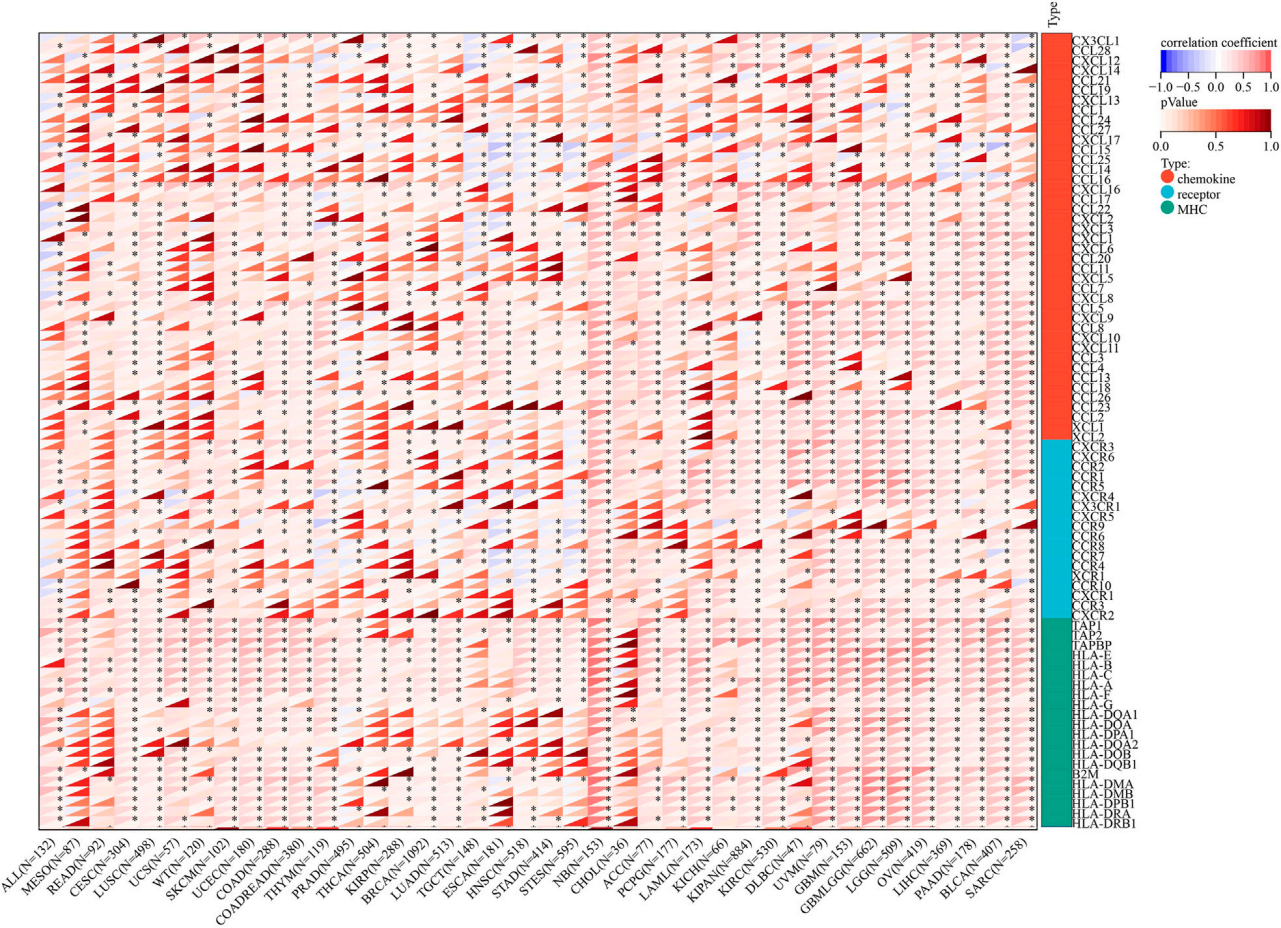
Analysis of correlation between the expression of AGTRAP and immunoregulatory genes.

Figure 12 shows that the immunoregulatory genes of CESC, LUSC, SKCM, COAD, THYM, STES, NB, ACC, PCPG, KIPAN, KIRC, DLBC, UVM, GBM, LGG, OV, LIHC, PAAD, BLCA, and SARC were highly related with AGTRAP. Importantly, MHC-related genes were more associated with AGTRAP expression than the chemokine-related and receptor-related genes in pan-cancer.

### Tumor mutational burden, microsatellite instability, neoantigen, and mismatch repair mutation correlation analysis

**FIGURE 13:**
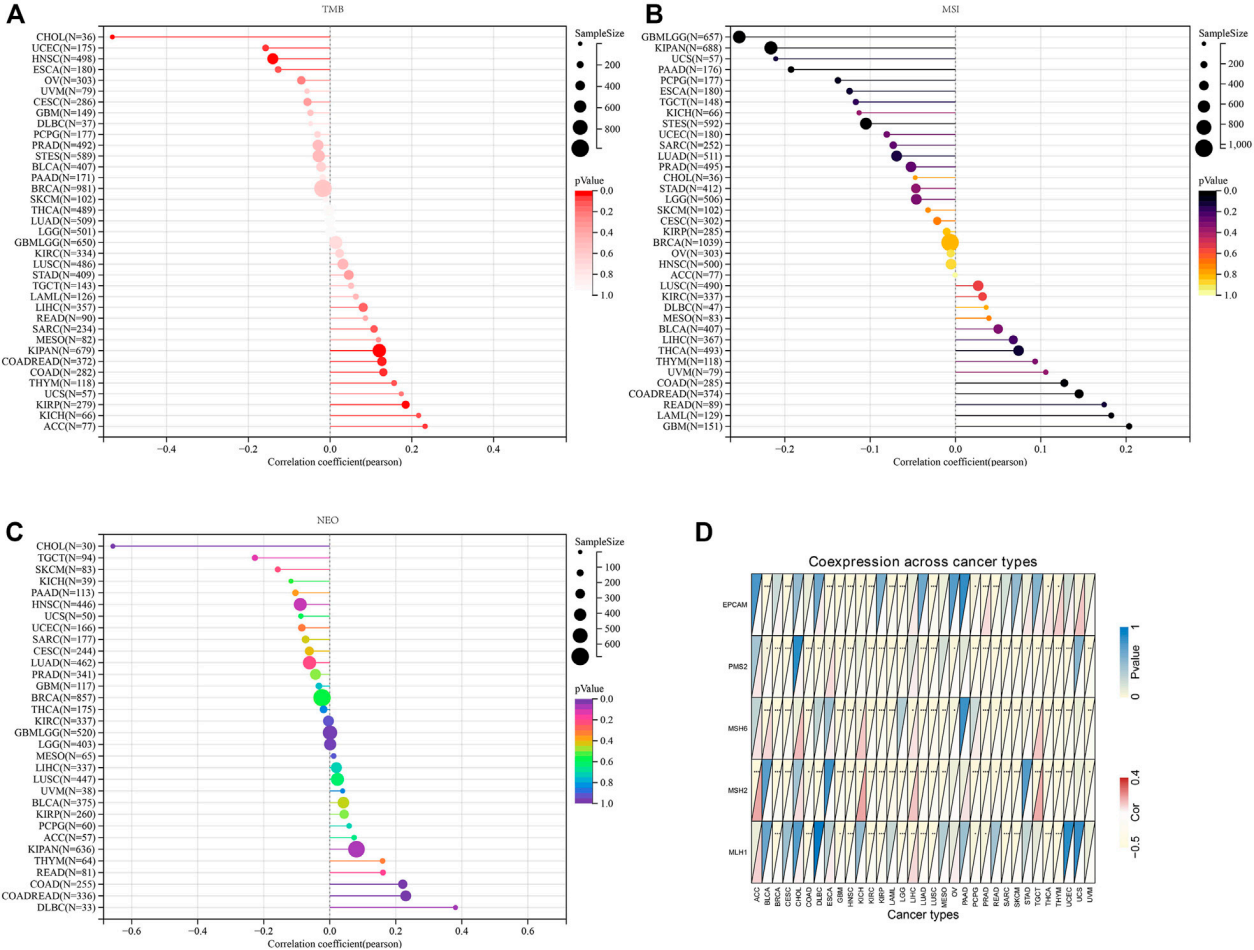
Analysis of relationship between the expression of AGTRAP and novel biomarkers in order to predict immunotherapy responses. **(A)** Correlation between the expression of AGTRAP and tumor mutation burden (TMB). **(B)** Correlation between the expression of AGTRAP and microsatellite instability (MSI). **(C)** Correlation between the expression of AGTRAP and neoantigen. **(D)** Correlation between the expression of AGTRAP and five mismatch repair (MMR) genes (EPCAM, PMS2, MSH6, MSH2, and MLH1).

TMB, MSI, and neoantigen are the three novel biomarkers related with immunotherapy responses. Correlation analyses were performed between these biomarkers and AGTRAP expression in pan-cancer. The AGTRAP expression level is significantly associated with TMB in CHOL (R < -0.2), ACC, and KICH (R > 0.2) (Figure 13A). Moreover, a significant correlation between AGTRAP and MSI is found in KIPAN and USC (R < -0.2), and GBM (R > 0.2) (Figure 13B). In addition, the relationship between AGTRAP and neoantigen was estimated, and CHOL and TGCT showed a negative correlation (R < -0.2), while DLBC and COAD showed a positive correlation (R > 0.2) (Figure 13C).

MMR genes play a vital role in maintaining gene stability, such as correcting base mismatches and deletions and insertion errors (Liu et al., 2017). Therefore, coexpression analysis of MMR genes and AGTRAP in pan-cancer was conducted and presented by the heatmap. Figure 13D shows that all five MMR genes we chose are significantly related with various cancers, in which PMS2, MSH6, and MSH2 are more related with AGTRAP than the other MMR genes. In addition, GBM, HNSC, LUSC, PRAD, THCA, and THYM are cancers in which AGTRAP expression is significantly related with all the five MMR genes.

### Immunohistochemistry of the angiotensin II receptor-associated protein in pan-cancer

**FIGURE 14:**
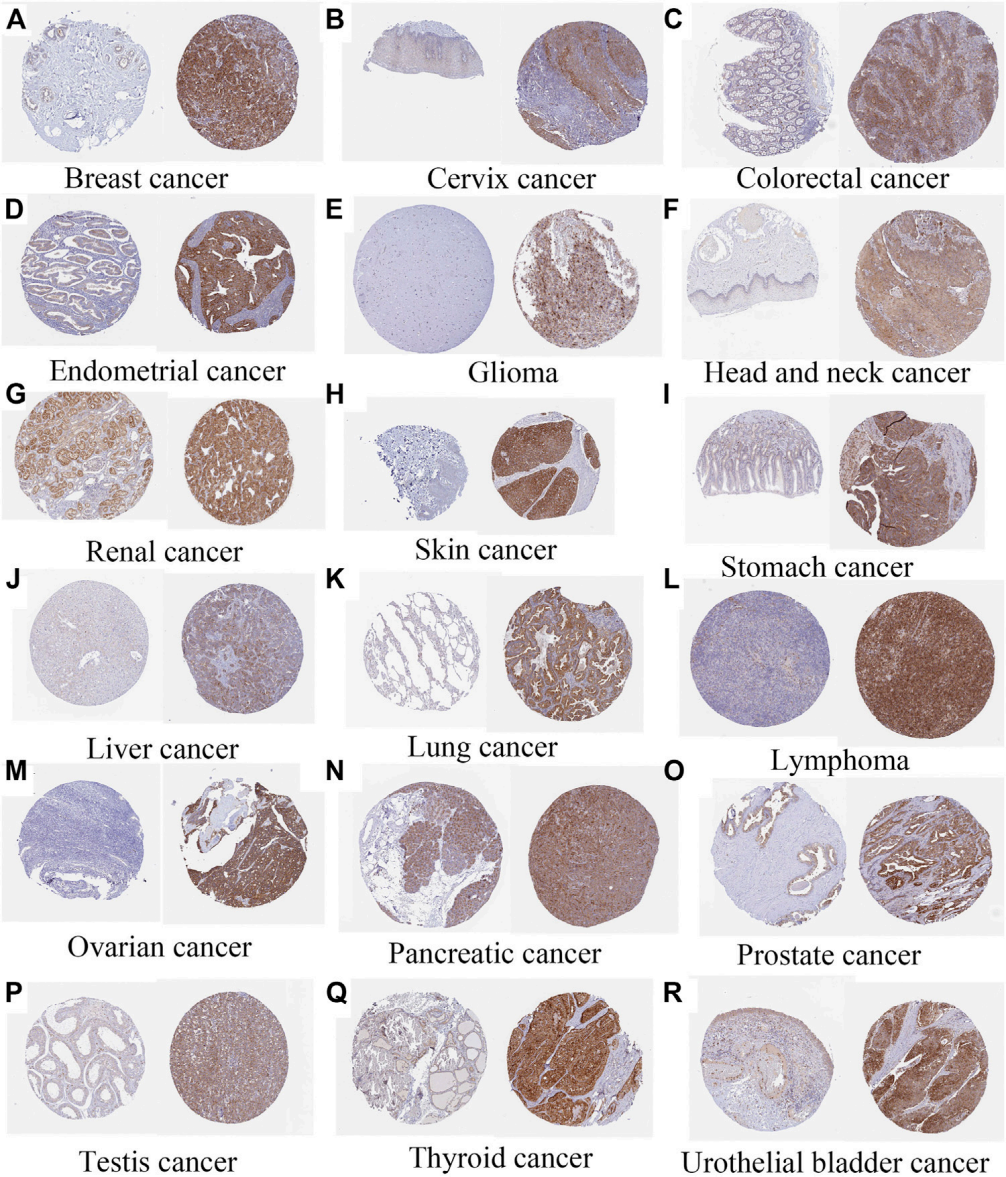
Immunohistochemistry of AGTRAP expression (normal tissue is on the left and tumor tissue is on the right) **(A)** Breast cancer (https://www.proteinatlas.org/ENSG00000177674-AGTRAP/pathology/breast+cancer). **(B)** Cervix cancer (https://www.proteinatlas.org/ENSG00000177674-AGTRAP/pathology/cervical+cancer). **(C)** Colorectal cancer (https://www.proteinatlas.org/ENSG00000177674-AGTRAP/pathology/colorectal+cancer). **(D)** Endometrial cancer (https://www.proteinatlas.org/ENSG00000177674-AGTRAP/pathology/endometrial+cancer). **(E)** Glioma (https://www.proteinatlas.org/ENSG00000177674-AGTRAP/pathology/glioma). **(F)** AGTRAP expression in head and neck cancer (https://www.proteinatlas.org/ENSG00000177674-AGTRAP/pathology/head+and+neck+cancer). **(G)** Renal cancer (https://www.proteinatlas.org/ENSG00000177674-AGTRAP/pathology/renal+cancer). **(H)** Skin cancer (https://www.proteinatlas.org/ENSG00000177674-AGTRAP/pathology/skin+cancer). **(I)** Stomach cancer (https://www.proteinatlas.org/ENSG00000177674-AGTRAP/pathology/stomach+cancer). **(J)** Liver cancer (https://www.proteinatlas.org/ENSG00000177674-AGTRAP/pathology/liver+cancer). **(K)** Lung cancer (https://www.proteinatlas.org/ENSG00000177674-AGTRAP/pathology/lung+cancer). **(L)** Lymphoma (https://www.proteinatlas.org/ENSG00000177674-AGTRAP/pathology/lymphoma). **(M)** Ovarian cancer (https://www.proteinatlas.org/ENSG00000177674-AGTRAP/pathology/ovarian+cancer). **(N)** Pancreatic cancer (https://www.proteinatlas.org/ENSG00000177674-AGTRAP/pathology/pancreatic+cancer). **(O)** Prostate cancer (https://www.proteinatlas.org/ENSG00000177674-AGTRAP/pathology/prostate+cancer). **(P)** Testis cancer (https://www.proteinatlas.org/ENSG00000177674-AGTRAP/pathology/testis+cancer). **(Q)** Thyroid cancer (https://www.proteinatlas.org/ENSG00000177674-AGTRAP/pathology/thyroid+cancer). **(R)** Urothelial bladder cancer (https://www.proteinatlas.org/ENSG00000177674-AGTRAP/pathology/urothelial+cancer).

Immunohistochemistry analysis highlighted the expression of AGTRAP in breast cancer, cervix cancer, colorectal cancer, endometrial cancer, glioma, head and neck cancer, liver cancer, lung cancer, lymphoma, melanoma, ovarian cancer, pancreatic cancer, prostate cancer, renal cancer, skin cancer, stomach cancer, testis cancer, thyroid cancer, and urothelial cancer (Figure 14).

### qRT-PCR of the angiotensin II receptor-associated protein in pan-cancer

To further verify the abnormal expression of AGTRAP in pan-cancer, we conducted qRT-PCR to detect the mRNA expression in breast cancer, pancreatic cancer, and gastric cancer. The results showed that AGTRAP is significantly highly expressed in breast cancer, pancreatic cancer, and gastric cancer, which verifies our bioinformatic analysis (Supplementary Figure S1).

### Western blot of the angiotensin II receptor-associated protein and the signaling pathway in pan-cancer

In addition, we performed Western blot in pan-cancer. Subsequently, high expression of AGTRAP was identified in breast cancer, pancreatic cancer, and gastric cancer (Supplementary Figure S2A). To explore the mechanism underlying AGTRAP, the protein expression of AKT/mTOR axis was measured by Western blot. The results showed that p-AKT and p-mTOR expression of si-AGTRAP was significantly lower than the expression of control and si-NC (Supplementary Figure S2B).

## Discussion

As a protein that regulates visceral obesity-related metabolism, AGTRAP has been widely researched in metabolic disorders (Maeda et al., 2013). Accumulating evidence confirmed that AT1R can regulate the RAS signaling pathway, thereby affecting the handling of renal sodium (Tamura et al., 2022). The overexpression of AT1R would excessively activate the RAS signaling, leading to the retention of sodium which is related to hypertension (Arendse et al., 2019). As a binding protein of the carboxyl-terminal domain, the AT1R-associated protein can suppress the hyperactivation of AT1R (Tamura et al., 2013). Previous studies confirmed that AT1R-associated proteins can inhibit the angiotensin-dependent hypertension by decreasing the sodium reabsorption (Ohsawa et al., 2014). In addition to metabolic functions, several bioinformatic analyses demonstrated that AGTRAP can be used as a component of prognostic signatures in brain lower grade glioma, tongue squamous cell carcinomas, and melanoma, revealing the potential of prognostic ability of AGTRAP (Loftus et al., 2017; Zeng et al., 2019; Xiong et al., 2020). However, details of AGTRAP about expression, survival, mechanism, and treatment response in diverse cancers are highly unclear.

In our study, the high expression of AGTRAP was found in 14 kinds of cancer and low expression in 1 cancer type from TCGA datasets. Moreover, the analysis of combination of TCGA and GTEx datasets suggested that AGTRAP was highly expressed in 26 kinds of cancer and lowly expressed in six cancer types. In addition, the abnormal protein expression of AGTRAP has been confirmed in the CPTAC samples across breast cancer, colon cancer, head and neck cancer, pancreatic cancer, glioblastoma multiforme, and hepatocellular carcinoma. Survival analysis showed that 12 kinds of cancers have poorer OS significantly related with the overexpression of AGTRAP and 2 types of cancers have better OS associated with overexpressed AGTRAP. According to our results, we found that our study is consistent with the past studies which researched AGTRAP in glioma and hepatocellular carcinoma (Xiong et al., 2020; Liu et al., 2021). However, in our study, the high expression of AGTRAP in colon cancer showed better survival which is contrary to a previous study (Sanz-Pamplona et al., 2016). In view of the deficiency of studies of AGTRAP in cancers, relevant research studies are urgently needed.

The PPI network revealed the most related 10 genes of AGTRAP, namely, AGTR1, JAK2, RAF1, CAMK2A, CAMK2B, CAMK2D, CAMK2G, CLCN6, ARAF, and PITPNC1. As shown in Table 1, most of them have been confirmed to influence tumor progression in various cancers, despite some genes showing contrary functions in different cancers. Based on our KEGG analysis, AGTRAP is related with protein processing in ER also called unfolded protein response (UPR) or endoplasmic reticulum (ER) stress (Hetz and Saxena, 2017; Hetz et al., 2020). ER stress can degrade misfolded proteins, decrease protein translation, and induce apoptosis (Wang et al., 2017; You et al., 2021). Researchers have reported that CHOP, Bcl-2 family, caspase-12, and JNK were components of ER stress related to the apoptotic pathway, whose cancer-related mechanisms have been demonstrated in various cancers (Ashkenazi et al., 2017; Kim et al., 2018; Tan et al., 2006). Therefore, there might be some potential mechanisms between these molecules and AGTRAP in cancers that need to be further explored. Furthermore, alcoholism is another related pathway of AGTRAP in cancer progression. From past studies, associations between alcoholism and various kinds of cancer have been widely explored. Lin et al. (2020) found that longer alcoholism history is related with the higher risk of colorectal cancer. In the study of Sun et al. (2020), an anti-alcoholism drug disulfiram showed the effect of causing immunogenic cell death in radiation-resistant breast cancer stem cells. Interestingly, Zhang et al. (2019) supported that disulfiram can induce autophagy-dependent apoptosis of pancreatic and breast cancer cells by evoking ER stress, which is accomplished through activation of IRE1α. Therefore, there might be some synergetic effects between ER stress and alcoholism, through which AGTRAP plays its role in cancerous development. Moreover, our analysis showed the relationship between AGTRAP and endocytosis. As a method of capturing materials from the extracellular environment, there takes place a special endocytic process named macropinocytosis, which can degrade protein to supply amino acids for cancer cell development and remove damaged material from the plasma membrane to restore membrane integrity (Commisso et al., 2013). Notably, it is this function of catching materials that makes it possible to mediate targeting therapy (Xiao et al., 2021). Nanomedicines (NMs) can effectively increase therapeutic efficacy and decrease toxicity for cancer therapy. Through different endocytosis processes, NMs can enter cells (Patel et al., 2019; Wang et al., 2020). If it is possible that drugs can selectively enter into cancer cells instead of delivering into healthy cells, cancer treatment would be highly efficient with low toxicity. In light of the KEGG analysis, AGTRAP may have potential mechanisms related with endocytosis in cancers, perhaps mediating the endocytosis process, which means that it is likely to use AGTRAP to reveal selectivity of drugs in cancer cells, which is worthy to be explored. In addition, AGTRAP is associated with the prostate cancer pathway according to enrichment analysis, which is consistent with our expression analysis that AGTRAP is highly expressed in prostate cancer. We also found the correlation between AGTRAP and androgen response, and since prostate cancer is strongly related with androgen, we believe that our analysis revealed the possible connection between AGTRAP and prostate cancer. According to the GO analysis, the most related biological process of AGTRAP is intracellular transport, which plays a role in direct movement of materials within cells (Clark, 2020). In addition, as the highest related molecular function of AGTRAP, transporter activity including lipid transporter activity, protein transporter activity, and transmembrane transporter activity have the function of enabling the directed movement of substances (Fuji et al., 2009; Nishimura and Stefan, 2020). Furthermore, combining the cellular component result, in which ER is the most enriched annotation, we inferred that AGTRAP is possible to influence cancer progression through ER-related material transportation. In our GSEA, we demonstrated that AGTRAP enriched in many immune-related pathways: “natural killer cell-mediated cytotoxicity pathway,” “chemokine signaling pathway,” “RIG-I-like receptor signaling pathway,” “toll-like receptor signaling pathway,” and “JAK-STAT signaling pathway”; and some metabolism-related pathways: “taurine and hypotaurine metabolism pathway” and “glycine, serine, and threonine metabolism pathway.” Evidence has confirmed that natural killer (NK) cells play a vital role in immunosurveillance and immunoediting, correlating with other immunity and assisting immunotherapy (Malmberg et al., 2017; Pugh-Toole et al., 2022). Veneziani et al. (2022) found that the function of NK cells can be strengthened by toll-like receptor 8 agonists, meaning that the interaction between NK cells and toll-like receptor signaling pathway, both of which are potential pathways that AGTRAP acts on. In addition, the enrichment of the chemokine signaling pathway was consisted in the abundant correlation between immunomodulators and AGTRAP, meaning that AGTRAP has functions in regulating immunomodulators, promoting tumor progression. In addition, RIG-I-like receptor and toll-like receptor are two nucleic acid sensors whose actions for innate immunity and potential for immunotherapy have been identified (Kanzler et al., 2007; Loo and Gale, 2011). Furthermore, previous studies proved that taurine, serine–glycine, and threonine metabolism could promote tumorigenesis and cancer cell proliferation in various kinds of cancers through diverse pathways (Locasale, 2013; Kim et al., 2014; Lin et al., 2018). In conclusion, our study indicated that AGTRAP might regulate these metabolism-related and immune-related pathways, leading to tumor development.

**TABLE 1:** AGTRAP-related genes.

Gene	Official full name	Ensemble	Function
AGTR1	Angiotensin II receptor type 1	ENSG00000144891	AGTR1 inhibits the progression of lung adenocarcinoma and promotes the proliferation of ovarian cancer
JAK2	Janus kinase 2	ENSG00000096968	JAK2 promotes the development and metastasis of lung adenocarcinoma and enhances osteosarcoma growth
RAF1	Raf-1 proto-oncogene	ENSG00000132155	RAF1 promotes progression and predicts poor survival of liver cancer; RAF1 amplification drives bladder tumorigenesis through activating the MAPK pathway
PITPNC1	Phosphatidylinositol transfer protein cytoplasmic 1	ENSG00000154217	PITPNC1 is overexpressed in metastatic breast, melanoma, and colon cancers and enhances vesicular secretion capacity in malignancy; PITPNC1-mediated fatty acid metabolic reprogramming promotes gastric cancer metastasis
ARAF	A-raf proto-oncogene	ENSG00000078061	ARAF promotes gall bladder tumorigenesis; ARAF mutation increases resistance of belvarafenib in melanoma
CLCN6	Chloride voltage-gated channel 6	ENSG00000011021	CLCN6 mutation causes West syndrome
CAMK2A	Calcium/calmodulin-dependent protein kinase II alpha	ENSG00000070808	CAMK2A promotes triple negative breast cancer metastasis, facilitates lung adenocarcinoma progression, and leads to poor prognosis
CAMK2B	Calcium/calmodulin-dependent protein kinase II beta	ENSG00000058404	CAMK2B inhibits papillary renal cell carcinoma and breast cancer proliferation
CAMK2D	Calcium/calmodulin-dependent protein kinase II delta	ENSG00000145349	CAMK2D increases resistance to cisplatin in ovarian cancer, suppresses gastric cancer progression and metastasis, and inhibits the growth of liver cancer
CAMK2G	Calcium/calmodulin-dependent protein kinase II gamma	ENSG00000148660	CAMK2G drives cisplatin resistance in ovarian cancer, promotes breast cancer progression and metastasis, and enhances lung tumorigenesis

A total of three novel markers used to predict immunotherapy-responses including TMB, MSI, and neoantigen were evaluated in pan-cancer. Immune checkpoint inhibitors (ICIs) have been demonstrated to be efficient in treating various kinds of cancers (Darvin et al., 2018). Currently, despite having found a lot of biomarkers for ICI and OS such as different immune checkpoints, PD-L1 is the only one which has been widely confirmed, such as the validated effectiveness of pembrolizumab for nonsmall cell lung cancer selected with PD-L1 > 50% and the combination of atezolizumab and nab-paclitaxel for metastatic triple negative breast cancer with PD-L1 > 1% (Schmid et al., 2018; Reck et al., 2019). Nevertheless, the prediction ability of PD-L1 is imperfect because of its heterogeneity and lability (Kim and Chung, 2019). Therefore, TMB, which is defined as the number of nonsynonymous exonic mutations per megabase, is used to predict the response of ICI (Fumet et al., 2020). Another biomarker, MSI-H, is demonstrated to have better prognoses in colon cancer patients (Latham et al., 2019). Moreover, neoantigens as mutant peptides are produced through antitumor immune response, which is elicited by somatic alterations where mutated peptide fragments are generated and then presented on the class I major histocompatibility complex (MHC-1) (Yarchoan et al., 2017). Since neoantigens can impel anticancer immunity and is tumor-specific, it has been regarded as potential therapeutic targets (Schumacher and Schreiber, 2015). Through our correlation analysis, the overexpression of AGTRAP predicts better immunotherapy responses in ACC, KICH, GBM, DLBC, and COAD. In view of the prediction ability of TMB, MSI, and neoantigen in diverse cancers, potential of AGTRAP for predicting drug responses combined with these novel biomarkers in pan-cancer is promising. Immune cells play important roles in tumorigenesis. In cancerous progression, immune cells can effectively eliminate tumor cells in early stages, but cancer cells gradually generate the tumor immune escape and influence the state of immune cells (Bremnes et al., 2011; Gonzalez et al., 2018). Among immune cells, neutrophils, natural killer cells, macrophages, dendritic cells, B cells, and T cells are those which are mostly related with cancer development. From our results, we found that correlations between AGTRAP expression and diverse immune cells can be found in almost all kinds of cancers. In addition, AGTRAP expression is significantly related with ImmuneScore in 19 types of cancers and with StromalScore in 17 types of cancers, which also reveals the relationship between AGTRAP and cancer immunity. Importantly, we found that AGTRAP expression was positively related with M2 macrophage infiltration among 25 cancer types. M2 macrophage is a kind of tumor-associated macrophages (TAMs) that play a vital role in forming TME (Pan et al., 2020). Previous research confirmed that M2 macrophage has functions of promoting angiogenesis, reconstructing tissue, repairing injury, and facilitating tumorigenesis and progression (Zhu et al., 2017; Annamalai et al., 2018). Chen et al. (2017) demonstrated that increased M2 macrophage was associated with poor prognosis and metastasis in breast cancer. Also, Yin et al. (2016) uncovered that M2 macrophage could promote spheroid formation and tumor growth stimulating metastasis in ovarian cancer. By combining previous studies and our analysis, we could speculate that AGTRAP might increase the M2 macrophage infiltration in TME which leads to tumor development. All of the factors we discussed previously have been used to evaluate the TME. As the biomarker for cancers, TME can be used to predict the responses to treatment and prognoses of patients. Based on our correlation analyses, AGTRAP shows the relationship with TME that makes the precise prediction for drug responses and outcomes possible.

In order to further reveal the mechanism of AGTRAP in influencing cancer progression, we analyzed the correlation between AGTRAP and some vital genes including immune checkpoint, RNA methylation, immunoregulator, and MMR. Studies have demonstrated that MMR plays vital roles in maintaining stability and integrity of the genome in cells (Li, 2008). Abnormality of MMR can augment the mutation frequency of related genes which would promote cancer growth and affect therapy responses (Green et al., 2011). The chemokine receptor, MHC, has been widely confirmed to increase tumorous growth and metastasis (Zlotnik et al., 2011; Nagarsheth et al., 2017; Miles et al., 2021). Bruchard et al. (2022) found that CCL20 can boost ILC3-dependent anticancer immunity and increase cancer sensitivity to immunotherapy. Dai et al. (2021) demonstrated the efficacy of CXCL13+CD8+T-cell infiltration for predicting the prognosis and being an immunotherapeutic target in clear cell renal cell carcinoma. RNA methylation, a common chemical modification in eukaryotic RNAs, is related with tumorous development acting through being catalyzed by writers, being removed by erasers, and interacting with readers (Lan et al., 2019). For instance, METTL14, a writer of m(6)A methylation, demonstrated downregulation in gastric cancer tissues and poor prognosis in gastric cancer patients (Fan et al., 2022). In addition, TRMT6/TRMT61A-mediated m(1)A methylation can drive liver tumorigenesis through cholesterol metabolism (Wang et al., 2021) and NSUN2-mediated and YBX1-mediated m(5)C methylation confirmed driving bladder carcinoma tumorigenesis (Chen et al., 2019). PD-l/PD-L1 is the most common immune checkpoint and of great significance for cancer, but others should not be ignored. Analyses of cancer-related genes combining with immune checkpoints have huge potentials for researching tumorigenesis and treatments. According to our study, AGTRAP shows great relationships with immune checkpoint, RNA methylation, immunoregulator, and MMR genes in pan-cancer, which means that there possibly exist some interactions between them influencing cancer progression. However, this field is almost blank and needs further exploration.

To further verify the protein expression of AGTRAP in pan-cancer, Western blot was also conducted on breast cancer, pancreatic cancer, and gastric cancer. Our results demonstrated that AGTRAP was highly expressed in these cancer types, which is consistent with the analysis across CPTAC dataset. Subsequently, the AKT/mTOR axis was detected both in breast cancer and gastric cancer. The results revealed that p-AKT and p-mTOR protein expression was significantly highly expressed, meaning that the AKT/mTOR axis was the possible downstream of AGTRAP. Previous studies reported that AKT and mTOR proteins function by phosphorylation (Wang et al., 2022; Zhu et al., 2022), and AGTRAP can promote phosphorylation of AKT and mTOR, initiating the AKT/mTOR axis. Accumulating evidence has demonstrated the functions of p-AKT and p-mTOR in promoting the progression of breast cancer and gastric cancer (Xu et al., 2021; Khan et al., 2022). Our study reveals that a novel biomarker, AGTRAP, can regulate the AKT/mTOR axis.

Nevertheless, the study still has some limitations. First, although with a global analysis for pan-cancer, details about each cancer type are insufficient. Thus, we will conduct deep analysis for specific cancer in our future studies. In addition, as a pan-cancer bioinformatic study, although with reasonable logic and comprehensive analysis for diverse cancers, combining with the verification of AGTRAP expression in three cancer types, deeper mechanisms of AGTRAP in each cancer type still need to be explored in the future.

In conclusion, our analysis is the first to explore the expression difference of AGTRAP between pan-cancer samples and their normal tissues, analyzing the prognostic impact and potential mechanisms of AGTRAP and its related genes in different kinds of cancers and revealing the correlation between AGTRAP and TME, TMB, MSI, neoantigen, and immune-related genes, which can assist predicting immunotherapy responses. In this study, we showed that AGTRAP and its related genes show abnormal expressions in different types of cancers that might affect tumor progression through regulating immune-related and metabolism-related pathways. Moreover, the abnormal expression of AGTRAP is associated with several immune-related biomarkers and could effectively predict drug responses, meaning that AGTRAP has the potential to become a target for antitumor immunotherapy.

## Data Availability

The datasets presented in this study can be found in online repositories. The names of the repository/repositories and accession number(s) can be found in the article/Supplementary Material.

## Glossary

AGTRAP: angiotensin II receptor-associated protein
ALL: acute lymphoblastic leukemia
AT1R: Ang II type 1 receptor
BLCA: bladder urothelial carcinoma
BRCA: breast invasive carcinoma
CESC: cervical squamous cell carcinoma and endo-cervical adenocarcinoma
CHOL: cholangiocarcinoma
CIs: confidence intervals
COAD: colon adenocarcinoma
CPTAC: Clinical Proteomic Tumor Analysis Consortium
DFS: disease-free survival
DSS: disease-specific survival
EPCAM: epithelial cell adhesion molecule
ER: endoplasmic reticulum
ESCA: esophageal carcinoma
FISH: fluorescence *in situ* hybridization
GBM: glioblastoma multiforme,
GEPIA2: Gene Expression Profiling Interactive Analysis, version 2
GO: Gene Ontology
GSEA: Gene set enrichment analysis
GTEx: Genotype-Tissue Expression
HNSC: head and neck squamous cell carcinoma
HRs: hazard ratios
ICIs: immune checkpoint inhibitors
KEGG: Kyoto Encyclopedia of Genes and Genomes
KICH: kidney chromophobe
KIPAN: Pan-kidney
KIRC: kidney renal clear cell carcinoma
KIRP: kidney renal papillary cell carcinoma
LAML: acute myeloid leukemia
LGG: brain lower grade glioma
LIHC: liver hepatocellular carcinoma
LUAD: lung adenocarcinoma
LUSC: lung squamous cell carcinoma
m(1)A: N1-methyladenosine
m(5)C: 5-methylcytosine
m(6)A: N6-methyladenosine
MAPK: mitogen-activated protein kinase
MESO: mesothelioma
MHC-1: Class I major histocompatibility complex
MLH1: MutL protein homolog 1
MMR: mismatch repair
MSH2: MutS protein homolog 2
MSH6: MutS homolog 6
MSI-H: high microsatellite instability
NB: neuroblastoma
NF-KB: factor-kappa B subunit 1
NK: natural killer
NMs: nanomedicines
OS: overall survival
OV: ovarian serous cystadenocarcinoma
PAAD: pancreatic adenocarcinoma
PCPG: pheochromocytoma and paraganglioma
PFS: progression-free survival
PMS2: PMS1 homolog 2
PPI: protein–protein interaction
PRAD: prostate adenocarcinoma
RAS: renin–angiotensin system
READ: rectum adenocarcinoma
SARC: sarcoma
SKCM: skin cutaneous melanoma
STAD: stomach adenocarcinoma
STES: stomach and esophageal carcinoma
TAMs: tumor-associated macrophages
TARGET: Therapeutically Applicable Research to Generate Effective Treatments
TCGA: The Cancer Genome Atlas
TCGA-ACC: The Cancer Genome Atlas Adrenocortical Carcinoma
TCGA-USC: The Cancer Genome Atlas Uterine Serous Carcinoma
TGCT: testicular germ cell tumors
THCA: thyroid carcinoma
THPA: The Human Protein Atlas
TILs: tumor-infiltrating lymphocytes
TIMER: Tumor Immune Estimation Resource
TMB: tumor burden
TME: tumor microenvironment
TPM: transcripts per million
UCEC: uterine corpus endometrial carcinoma
UCS: uterine carcinosarcoma
UCSC: University of California, Santa Cruz
UPR: unfolded protein response
UVM: uveal melanoma
WT: Wilms’ tumor
