# Supplementary figures and images for: Pan-cancer analysis of the angiotensin II receptor-associated protein as a prognostic and immunological gene predicting immunotherapy responses in pan-cancer
Source: Front Cell Dev Biol. 2022 Aug 19;10:913684. doi: 10.3389/fcell.2022.913684 (PMC9437438; doi:10.3389/fcell.2022.913684)

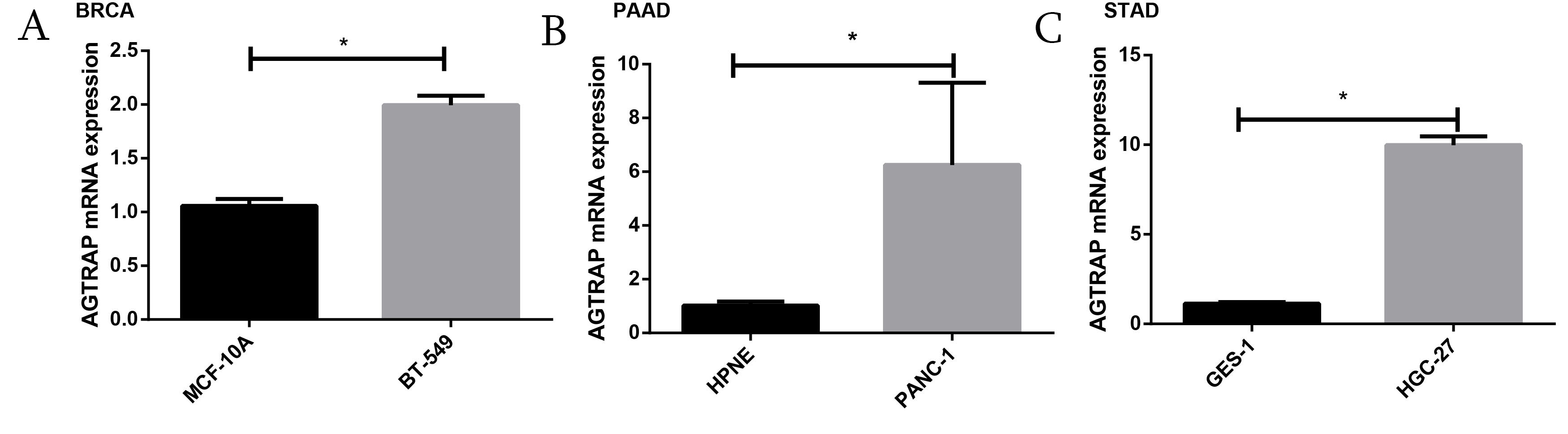

Supplement: Supplementary file 1 [file Image1.JPEG]

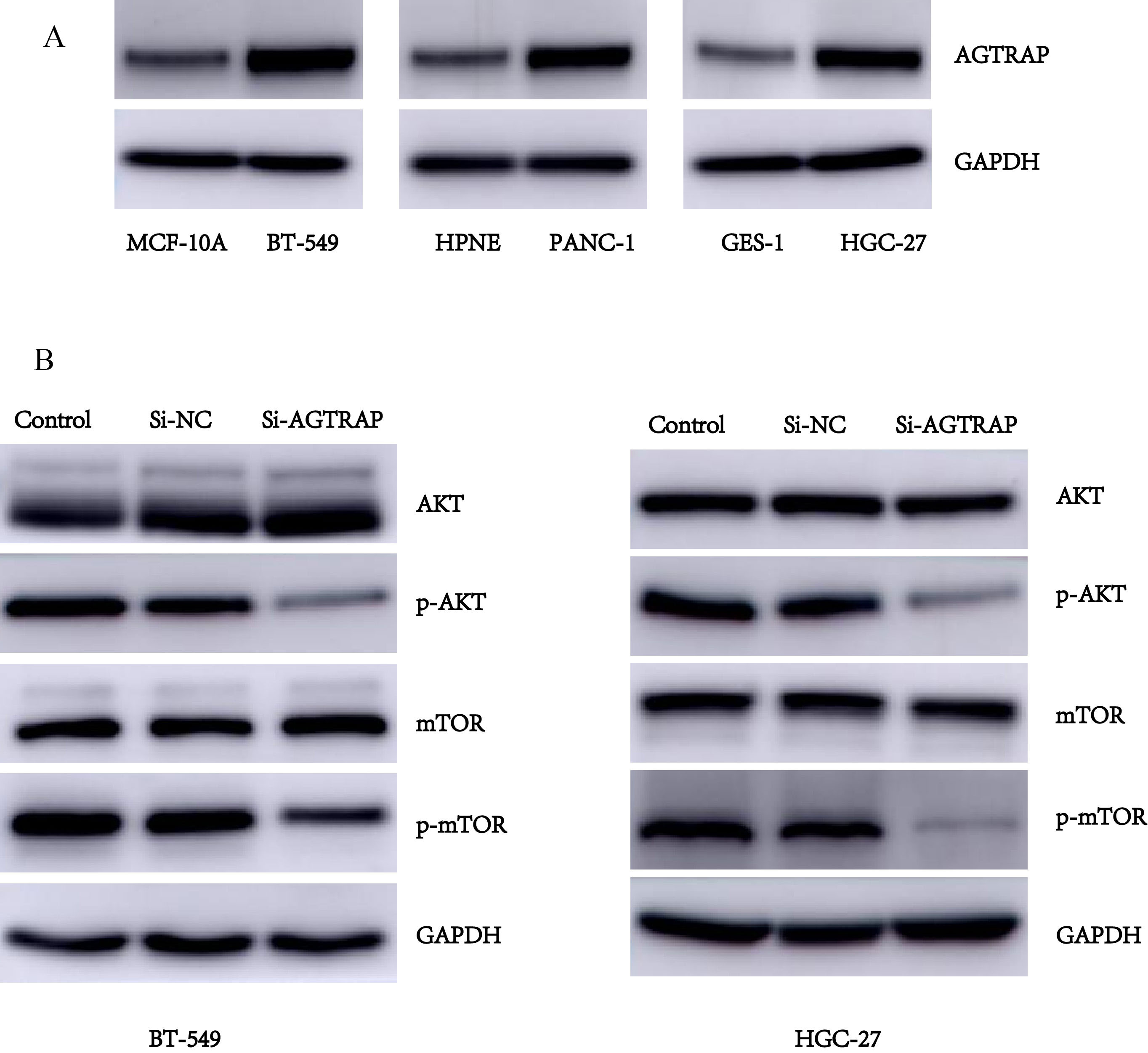

Supplement: Supplementary file 2 [file Image2.JPEG]
