# Supplementary material for: Pan-cancer analysis of the angiotensin II receptor-associated protein as a prognostic and immunological gene predicting immunotherapy responses in pan-cancer
Source: Front Cell Dev Biol. 2022 Aug 19;10:913684. doi: 10.3389/fcell.2022.913684 (PMC9437438; doi:10.3389/fcell.2022.913684)
Supplement: Supplementary file 3 [file DataSheet2.DOCX]

library(limma)

gene="AGTRAP"

files=dir()

files=grep("^symbol.", files, value=T)

geneList=list()

for(i in files){

CancerType=gsub("symbol\\.|\\.txt", "", i)

rt=read.table(i, header=T, sep="\t", check.names=F)

geneList[[CancerType]]=as.vector(rt[,1])

}

interGenes=Reduce(intersect, geneList)

outTab=data.frame()

allTab=data.frame()

j=0

for(i in files){

CancerType=gsub("symbol\\.|\\.txt", "", i)

rt=read.table(i, header=T, sep="\t", check.names=F)

rt=as.matrix(rt)

rownames(rt)=rt[,1]

exp=rt[,2:ncol(rt)]

dimnames=list(rownames(exp), colnames(exp))

data=matrix(as.numeric(as.matrix(exp)), nrow=nrow(exp), dimnames=dimnames)

data=t(avereps(data))

row.names(data)=gsub(".$", "", row.names(data))

data=t(avereps(data))

group=sapply(strsplit(colnames(data),"\\-"), "[", 4)

group=sapply(strsplit(group,""), "[", 1)

Type=ifelse(group==0, "Tumor", "Normal")

geneExp=t(data[gene,,drop=F])

outTab=rbind(outTab, cbind(geneExp,Type,CancerType))

j=j+1

if(j==1){

allTab=data[interGenes,group==0]

}else{

allTab=cbind(allTab, data[interGenes,group==0])

}

}

out=cbind(ID=row.names(outTab), outTab)

write.table(out, file="geneExp.txt", sep="\t", quote=F, row.names=F)

allTab=allTab[rowMeans(allTab)>0.1,]

allTab=cbind(ID=row.names(allTab), allTab)

write.table(allTab, file="merge.txt", sep="\t", quote=F, row.names=F)

library(plyr)

library(reshape2)

library(ggpubr)

inputFile="geneExp.txt"

setwd("C:\\biowolf\\panImmune\\06.diff")

data=read.table(inputFile, header=T, sep="\t", check.names=F)

gene=colnames(data)[2]

colnames(data)[2]="expression"

p=ggboxplot(data, x="CancerType", y="expression", color="Type",

xlab="",

ylab=paste0(gene," expression"),

width=0.6,

palette = c("blue","red") )

p=p+rotate_x_text(50)

p=p+stat_compare_means(aes(group=Type),

method="wilcox.test",

symnum.args=list(cutpoints = c(0, 0.001, 0.01, 0.05, 1), symbols = c("***", "**", "*", " ")),

label = "p.signif")

pdf(file="diff.pdf", width=8, height=5.5)

print(p)

dev.off()

data=data[(data[,"Type"]=="Tumor"),]

med=ddply(data,"CancerType",summarise,med=median(expression))

data$CancerType=factor(data$CancerType, levels=med[order(med[,"med"],decreasing = T), "CancerType"])

p=ggboxplot(data, x="CancerType", y="expression", fill="CancerType",

xlab="",

ylab=paste0(gene," expression"),

width=0.6,

#palette=rainbow(length(levels(factor(data$CancerType)))),

legend="")

pdf(file="boxplot.pdf", width=8, height=5.5)

p+rotate_x_text(50)

dev.off()

library(limma)

expFile="geneExp.txt"

setwd("C:\\biowolf\\panImmune\\10.preOS")

files=dir()

files=grep(".survival.tsv$", files, value=T)

surTime=data.frame()

for(surFile in files){

rt=read.table(surFile, header=T, sep="\t", check.names=F, row.names=1)

rt=rt[,c(3,1)]

surTime=rbind(surTime, rt)

}

colnames(surTime)=c("futime","fustat")

surTime=as.matrix(surTime)

row.names(surTime)=gsub(".$","",row.names(surTime))

exp=read.table(expFile, header=T, sep="\t", check.names=F, row.names=1)

exp=exp[(exp[,"Type"]=="Tumor"),]

sameSample=intersect(row.names(surTime), row.names(exp))

surTime=surTime[sameSample,,drop=F]

exp=exp[sameSample,,drop=F]

surData=cbind(surTime, exp)

surData=cbind(id=row.names(surData), surData)

write.table(surData, file="expTime.txt", sep="\t", quote=F, row.names=F)

library(survival)

library(survminer)

library(forestplot)

inputFile="expTime.txt"

setwd("G:")

rt=read.table(inputFile, header=T, sep="\t", check.names=F, row.names=1)

rt$futime=rt$futime/365

gene=colnames(rt)[3]

outTab=data.frame()

for(i in levels(factor(rt[,"CancerType"]))){

rt1=rt[(rt[,"CancerType"]==i),]

cox=coxph(Surv(futime, fustat) ~ rt1[,gene], data = rt1)

coxSummary = summary(cox)

coxP=coxSummary$coefficients[,"Pr(>|z|)"]

outTab=rbind(outTab,

cbind(cancer=i,

HR=coxSummary$conf.int[,"exp(coef)"],

HR.95L=coxSummary$conf.int[,"lower .95"],

HR.95H=coxSummary$conf.int[,"upper .95"],

pvalue=coxP) )

group=ifelse(rt1[,gene]>median(rt1[,gene]), "high", "low")

diff=survdiff(Surv(futime, fustat) ~ group, data=rt1)

pValue=1-pchisq(diff$chisq,df=1)

if(pValue<0.05){

if(pValue<0.001){

pValue="p<0.001"

}else{

pValue=paste0("p=",sprintf("%.03f",pValue))

}

fit=survfit(Surv(futime, fustat) ~ group, data = rt1)

surPlot=ggsurvplot(fit,

data=rt1,

title=paste0("Cancer: ",i),

pval=pValue,

pval.size=6,

conf.int=F,

legend.title=paste0(gene," levels"),

legend.labs=c("high","low"),

font.legend=12,

fontsize=4,

xlab="Time(years)",

ylab="Overall survival",

break.time.by = 2,

palette=c("red","blue"),

risk.table=TRUE,

risk.table.title="",

risk.table.height=.25)

pdf(file=paste0("survival.",i,".pdf"), width=6, height=5, onefile=FALSE)

print(surPlot)

dev.off()

}

}

write.table(outTab, file="cox.result.txt", sep="\t", row.names=F, quote=F)

bioForest=function(coxFile, forestFile, forestCol){

rt=read.table(coxFile,header=T,sep="\t",row.names=1,check.names=F)

data=as.matrix(rt)

HR=data[,1:3]

hr=sprintf("%.3f",HR[,"HR"])

hrLow=sprintf("%.3f",HR[,"HR.95L"])

hrHigh=sprintf("%.3f",HR[,"HR.95H"])

pVal=data[,"pvalue"]

pVal=ifelse(pVal<0.001, "<0.001", sprintf("%.3f", pVal))

clrs=fpColors(box=forestCol, line="darkblue", summary="royalblue")

tabletext <-

list(c(NA, rownames(HR)),

append("pvalue", pVal),

append("Hazard ratio",paste0(hr,"(",hrLow,"-",hrHigh,")")) )

pdf(file=forestFile, width=9, height=6, onefile=FALSE)

print(forestplot(tabletext,

rbind(rep(NA, 3), HR),

col=clrs,

graphwidth=unit(50, "mm"),

xlog=T,

lwd.ci=4,

boxsize=0.6,

title="Overall survival",

xlab="Hazard ratio",

txt_gp=fpTxtGp(ticks=gpar(cex=1.1),xlab=gpar(cex = 1.25))

))

dev.off()

}

bioForest(coxFile="cox.result.txt", forestFile="forest.pdf", forestCol="red")

library(limma)

library(survival)

library(survminer)

library(forestplot)

pFilter=0.05

expFile="geneExp.txt"

cliFile="Survival_SupplementalTable_S1_20171025_xena_sp"

setwd("G:\\")

bioForest=function(coxFile=null, forestFile=null, forestCol=null, titleName=null){

rt=read.table(coxFile, header=T, sep="\t", check.names=F, row.names=1)

data=as.matrix(rt)

HR=data[,1:3]

hr=sprintf("%.3f",HR[,"HR"])

hrLow=sprintf("%.3f",HR[,"HR.95L"])

hrHigh=sprintf("%.3f",HR[,"HR.95H"])

pVal=data[,"pvalue"]

pVal=ifelse(pVal<0.001, "<0.001", sprintf("%.3f", pVal))

clrs <- fpColors(box=forestCol,line="darkblue", summary="royalblue")

tabletext <-

list(c(NA, rownames(HR)),

append("pvalue", pVal),

append("Hazard ratio",paste0(hr,"(",hrLow,"-",hrHigh,")")) )

pdf(file=forestFile, width=9, height=6, onefile = FALSE)

forestplot(tabletext,

rbind(rep(NA, 3), HR),

col=clrs,

graphwidth=unit(50, "mm"),

xlog=T,

lwd.ci=4,

boxsize=0.6,

title=titleName,

xlab="Hazard ratio",

txt_gp=fpTxtGp(ticks=gpar(cex=1.1),xlab=gpar(cex = 1.25))

)

dev.off()

}

exp=read.table(expFile, header=T, sep="\t", check.names=F, row.names=1)

exp=exp[(exp[,"Type"]=="Tumor"),]

gene=colnames(exp)[1]

cli=read.table(cliFile, header=T, sep="\t", check.names=F, row.names=1)

cli=cli[,c("DFI.time","DFI","DSS.time","DSS","PFI.time","PFI")]

sameSample=intersect(row.names(cli), row.names(exp))

data=cbind(cli[sameSample,,drop=F], exp[sameSample,,drop=F])

cols=c("#EEEE00", "#00DD00", "#FF00FF")

project=c("DFS", "DSS", "PFS")

showNames=c("Disease free survival", "Disease specific survival", "Progression free survival")

for(i in 1:3){

k=2*i-1

rt=data[,c(k,k+1,7:ncol(data))]

rt=na.omit(rt)

colnames(rt)[1:3]=c("futime","fustat","gene")

rt$futime=rt$futime/365

outTab=data.frame()

for(cancerType in levels(factor(rt[,"CancerType"]))){

rt1=rt[(rt[,"CancerType"]==cancerType),]

if(nrow(rt1)<3){next}

group=ifelse(rt1[,"gene"]>median(rt1[,"gene"]), "high", "low")

diff=survdiff(Surv(futime, fustat) ~group,data = rt1)

pValue=1-pchisq(diff$chisq,df=1)

if(pValue<pFilter){

if(pValue<0.001){

pValue="p<0.001"

}else{

pValue=paste0("p=",sprintf("%.03f",pValue))

}

fit <- survfit(Surv(futime, fustat) ~ group, data = rt1)

surPlot=ggsurvplot(fit,

data=rt1,

title=paste0("Cancer: ",cancerType),

pval=pValue,

pval.size=6,

conf.int=F,

legend.title=paste0(gene," levels"),

legend.labs=c("high","low"),

font.legend=12,

fontsize=4,

xlab="Time(years)",

ylab=showNames[i],

break.time.by=2,

palette=c("red","blue"),

risk.table=TRUE,

risk.table.title="",

risk.table.height=.25)

pdf(file=paste0(project[i],"_",cancerType,".pdf"), onefile=FALSE, width=6, height=5)

print(surPlot)

dev.off()

}

cox=coxph(Surv(futime, fustat) ~ gene, data = rt1)

coxSummary=summary(cox)

coxP=coxSummary$coefficients[,"Pr(>|z|)"]

outTab=rbind(outTab,

cbind(cancer=cancerType,

HR=coxSummary$conf.int[,"exp(coef)"],

HR.95L=coxSummary$conf.int[,"lower .95"],

HR.95H=coxSummary$conf.int[,"upper .95"],

pvalue=coxP) )

}

write.table(outTab,file=paste0(project[i],".cox.txt"),sep="\t",row.names=F,quote=F)

bioForest(coxFile=paste0(project[i],".cox.txt"),

forestFile=paste0(project[i],".forest.pdf"),

forestCol=cols[i],

titleName=showNames[i])

}

library(limma)

library(estimate)

setwd("C:\\")

files=dir()

files=grep("^symbol.", files, value=T)

outTab=data.frame()

for(i in files){

CancerType=gsub("symbol\\.|\\.txt", "", i)

rt=read.table(i, header=T, sep="\t", check.names=F)

rt=as.matrix(rt)

rownames(rt)=rt[,1]

exp=rt[,2:ncol(rt)]

dimnames=list(rownames(exp),colnames(exp))

data=matrix(as.numeric(as.matrix(exp)), nrow=nrow(exp), dimnames=dimnames)

data=t(avereps(data))

row.names(data)=gsub(".$", "", row.names(data))

data=t(avereps(data))

group=sapply(strsplit(colnames(data),"\\-"), "[", 4)

group=sapply(strsplit(group,""), "[", 1)

group=gsub("2", "1", group)

data=data[,group==0]

out=data[rowMeans(data)>0,]

out=rbind(ID=colnames(out), out)

write.table(out, file="uniq.symbol.txt", sep="\t", quote=F, col.names=F)

filterCommonGenes(input.f="uniq.symbol.txt", output.f="commonGenes.gct", id="GeneSymbol")

estimateScore(input.ds="commonGenes.gct", output.ds="estimateScore.gct")

scores=read.table("estimateScore.gct", header=T, skip=2)

rownames(scores)=scores[,1]

scores=t(scores[,3:ncol(scores)])

rownames(scores)=gsub("\\.", "\\-", rownames(scores))

outTab=rbind(outTab,cbind(scores, CancerType))

file.remove("uniq.symbol.txt")

file.remove("commonGenes.gct")

file.remove("estimateScore.gct")

}

out=cbind(ID=row.names(outTab), outTab)

write.table(out, file="estimateScores.txt", sep="\t", quote=F, row.names=F)

library(ggplot2)

library(ggpubr)

library(ggExtra)

corFilter=0.5

pFilter=0.05

expFile="geneExp.txt"

immFile="CIBERSORT.result.txt"

setwd("C:\\")

exp=read.table(expFile, header=T,sep="\t", check.names=F, row.names=1)

exp=exp[(exp[,"Type"]=="Tumor"),]

gene=colnames(exp)[1]

immune=read.table(immFile, header=T, sep="\t", check.names=F, row.names=1)

sameSample=intersect(row.names(immune), row.names(exp))

immune=immune[sameSample,]

exp=exp[sameSample,]

outTab=data.frame()

for(i in levels(factor(exp[,"CancerType"]))){

exp1=exp[(exp[,"CancerType"]==i),]

immune1=immune[(immune[,"CancerType"]==i),]

y=as.numeric(exp1[,1])

outVector=data.frame(i, gene)

for(j in colnames(immune1)[1:22]){

x=as.numeric(immune1[,j])

if(sd(x)>0.01){

df1=as.data.frame(cbind(x,y))

corT=cor.test(x,y,method="spearman")

cor=corT$estimate

pValue=corT$p.value

outVector=cbind(outVector,pValue)

if((abs(cor)>corFilter) & (pValue<pFilter)){

p1=ggplot(df1, aes(x, y)) +

xlab(j)+ylab(gene)+

ggtitle(paste0("\nCancer: ",i))+theme(title=element_text(size=10))+

geom_point()+ geom_smooth(method="lm", formula=y ~ x) + theme_bw()+

stat_cor(method = 'spearman', aes(x =x, y =y))

p2=ggMarginal(p1, type = "density", xparams = list(fill = "orange"),yparams = list(fill = "blue"))

pdf(file=paste0("estimateCor.",i,"_",j,".pdf"), width=5, height=5.1)

print(p2)

dev.off()

}

}

else{

outVector=cbind(outVector,pValue=1)

}

}

outTab=rbind(outTab, outVector)

}

colNames=c("CancerType", "Gene", colnames(immune)[1:22])

colnames(outTab)=colNames

write.table(outTab, file="immuneCor.result.txt", sep="\t", row.names=F, quote=F)

library(limma)

library(org.Hs.eg.db)

library(clusterProfiler)

library(enrichplot)

gene="AGTRAP"

gmtFile="c2.cp.kegg.v7.4.symbols.gmt"

setwd("C:\\")

gmt=read.gmt(gmtFile)

files=dir()

files=grep("^symbol.", files, value=T)

for(i in files){

rt=read.table(i, header=T, sep="\t", check.names=F)

CancerType=gsub("^symbol\\.|\\.txt$", "", i)

rt=as.matrix(rt)

rownames(rt)=rt[,1]

exp=rt[,2:ncol(rt)]

dimnames=list(rownames(exp), colnames(exp))

data=matrix(as.numeric(as.matrix(exp)), nrow=nrow(exp), dimnames=dimnames)

data=avereps(data)

group=sapply(strsplit(colnames(data),"\\-"), "[", 4)

group=sapply(strsplit(group,""), "[", 1)

group=gsub("2", "1", group)

data=data[,group==0]

dataL=data[,(data[gene,]<=median(data[gene,]))]

dataH=data[,(data[gene,]>median(data[gene,]))]

meanL=rowMeans(dataL)

meanH=rowMeans(dataH)

meanL[meanL<0.00001]=0.00001

meanH[meanH<0.00001]=0.00001

logFC=log2(meanH/meanL)

logFC=sort(logFC, decreasing=T)

kk=GSEA(logFC, TERM2GENE=gmt, nPerm=100, pvalueCutoff=1)

kkTab=as.data.frame(kk)

kkTab=kkTab[kkTab$pvalue<0.05,]

write.table(kkTab, file=paste0("Term.",CancerType,".txt"), sep="\t", quote=F, row.names=F)

termNum=5

if(nrow(kkTab)>=termNum){

gseaplot=gseaplot2(kk,

row.names(kkTab)[1:termNum],

base_size=8,

title=paste0("Cancer: ",CancerType))

pdf(file=paste0("Term.",CancerType,".pdf"), width=8, height=6)

print(gseaplot)

dev.off()

}else if(nrow(kkTab)>0){

gseaplot=gseaplot2(kk,

row.names(kkTab),

base_size=8,

title=paste0("Cancer: ",CancerType))

pdf(file=paste0("Term.",CancerType,".pdf"), width=8, height=6)

print(gseaplot)

dev.off()

}

}

library(fmsb)

expFile="geneExp.txt"

tmbFile="TMB.txt"

col="red"

setwd("C:\\")

exp=read.table(expFile, header=T, sep="\t", check.names=F, row.names=1)

exp=exp[(exp[,"Type"]=="Tumor"),]

TMB=read.table(tmbFile, header=T, sep="\t", check.names=F, row.names=1)

TMB=as.matrix(TMB)

row.names(TMB)=gsub(".$", "", row.names(TMB))

sameSample=intersect(row.names(TMB), row.names(exp))

TMB=TMB[sameSample,]

exp=exp[sameSample,]

outTab=data.frame()

fmsbTab=data.frame()

for(i in levels(factor(exp[,"CancerType"]))){

exp1=exp[(exp[,"CancerType"]==i),]

TMB1=TMB[(TMB[,"CancerType"]==i),]

x=as.numeric(TMB1[,1])

y=as.numeric(exp1[,1])

corT=cor.test(x,y,method="spearman")

cor=corT$estimate

pValue=corT$p.value

sig=ifelse(pValue<0.001,"***",ifelse(pValue<0.01,"**",ifelse(pValue<0.05,"*"," ")))

outTab=rbind(outTab, cbind(CancerType=i,cor=cor,pValue=pValue))

fmsbTab=rbind(fmsbTab, cbind(CancerType=paste0(i,sig),cor=cor))

}

write.table(outTab,file="cor.result.txt",sep="\t",row.names=F,quote=F)

write.table(t(fmsbTab),file="fmsbInput.txt",sep="\t",col.names=F,quote=F)

data=read.table("fmsbInput.txt", header=T, sep="\t", check.names=F, row.names=1)

maxValue=ceiling(max(abs(data))*10)/10

data=rbind(rep(maxValue,ncol(data)),rep(-maxValue,ncol(data)),data)

pdf(file="radar.pdf", width=7, height=7)

radarchart(data, axistype=1,

title="Tumor mutation burden",

pcol=col,

plwd=2 ,

plty=1,

cglcol="grey",

cglty=1,

caxislabels=seq(-maxValue,maxValue,maxValue/2),

cglwd=1.2,

axislabcol="blue",

vlcex=0.8

)

dev.off()
