# Supplementary material for: Pan-cancer analysis of the angiotensin II receptor-associated protein as a prognostic and immunological gene predicting immunotherapy responses in pan-cancer
Source: Front Cell Dev Biol. 2022 Aug 19;10:913684. doi: 10.3389/fcell.2022.913684 (PMC9437438; doi:10.3389/fcell.2022.913684)
Supplement: Supplementary file 4 [file DataSheet1.DOCX]

Gene Symbol Gene ID PCC

LINC01137 ENSG00000233621.1 0.49

CORO1B ENSG00000172725.13 0.44

YIPF1 ENSG00000058799.13 0.43

RP11-545E17.3 ENSG00000223478.1 0.43

TMEM134 ENSG00000172663.8 0.43

RABEP2 ENSG00000177548.12 0.42

KLK3 ENSG00000142515.14 0.42

NANS ENSG00000095380.10 0.42

SDF4 ENSG00000078808.16 0.42

KLK2 ENSG00000167751.12 0.42

SPDEF ENSG00000124664.10 0.42

TMEM79 ENSG00000163472.18 0.41

HOXB13 ENSG00000159184.7 0.41

PIEZO1 ENSG00000103335.19 0.41

TMED3 ENSG00000166557.12 0.41

OAZ3 ENSG00000143450.14 0.41

NKX3-1 ENSG00000167034.9 0.40

TRPM4 ENSG00000130529.15 0.40

KLK4 ENSG00000167749.11 0.39

COPG1 ENSG00000181789.14 0.39

RP11-66B24.2 ENSG00000232386.9 0.39

GMPPB ENSG00000173540.12 0.39

TRPV6 ENSG00000165125.17 0.39

SLC45A3 ENSG00000158715.5 0.39

SYTL1 ENSG00000142765.17 0.39

FBXO6 ENSG00000116663.10 0.38

MRPL20 ENSG00000242485.5 0.38

UBE2J2 ENSG00000160087.20 0.38

RDH11 ENSG00000072042.12 0.38

RP11-108P20.4 ENSG00000267593.1 0.38

GNB2 ENSG00000172354.9 0.38

TSPAN1 ENSG00000117472.9 0.38

LDLRAP1 ENSG00000157978.11 0.38

GPN2 ENSG00000142751.14 0.38

MINOS1 ENSG00000173436.13 0.38

TMCO4 ENSG00000162542.13 0.38

LENG9 ENSG00000275183.1 0.38

ACPP ENSG00000014257.15 0.38

FAAH ENSG00000117480.15 0.37

STEAP2 ENSG00000157214.13 0.37

H2AFJ ENSG00000246705.4 0.37

STEAP1 ENSG00000164647.8 0.37

EIF3I ENSG00000084623.11 0.37

HMG20B ENSG00000064961.18 0.37

SLC30A4 ENSG00000104154.6 0.37

FLJ20021 ENSG00000254531.1 0.37

NMRAL1 ENSG00000153406.13 0.37

LYPLA2 ENSG00000011009.10 0.37

GRN ENSG00000030582.16 0.37

TMEM50A ENSG00000183726.10 0.36

BAIAP2 ENSG00000175866.15 0.36

GTF3C1 ENSG00000077235.17 0.36

NAAA ENSG00000138744.14 0.36

OR51E2 ENSG00000167332.7 0.36

RPS10L ENSG00000101278.6 0.36

SSU72 ENSG00000160075.11 0.36

RAB1B ENSG00000174903.14 0.36

HDLBP ENSG00000115677.16 0.36

TIGD6 ENSG00000164296.6 0.36

TMED9 ENSG00000184840.11 0.36

HOMER2 ENSG00000103942.12 0.36

NKILA ENSG00000278709.1 0.36

MAN1B1 ENSG00000177239.14 0.35

TMPRSS2 ENSG00000184012.11 0.35

ADPRHL2 ENSG00000116863.10 0.35

CHRNA2 ENSG00000120903.10 0.35

GMPPA ENSG00000144591.17 0.35

RCAN3 ENSG00000117602.11 0.35

CREB3L4 ENSG00000143578.15 0.35

PRAC2 ENSG00000229637.3 0.35

LMAN1L ENSG00000140506.16 0.35

AP2S1 ENSG00000042753.11 0.35

B3GALT6 ENSG00000176022.4 0.35

OR51A9P ENSG00000180723.6 0.35

RER1 ENSG00000157916.18 0.35

ANO7 ENSG00000146205.13 0.35

PACSIN3 ENSG00000165912.15 0.35

TSPO ENSG00000100300.17 0.35

NOC2L ENSG00000188976.10 0.35

DDOST ENSG00000244038.9 0.35

PEX14 ENSG00000142655.12 0.34

CHTF8 ENSG00000168802.12 0.34

CD151 ENSG00000177697.17 0.34

CAPZB ENSG00000077549.17 0.34

CTC-510F12.6 ENSG00000267576.1 0.34

SEC61A1 ENSG00000058262.9 0.34

TRGC1 ENSG00000211689.6 0.34

APRT ENSG00000198931.10 0.34

CAPNS1 ENSG00000126247.10 0.34

CPNE4 ENSG00000196353.11 0.34

MFSD10 ENSG00000109736.14 0.34

ORMDL2 ENSG00000123353.9 0.34

RPS6KA1 ENSG00000117676.13 0.34

PRAC1 ENSG00000159182.4 0.34

ZNF761 ENSG00000160336.14 0.34

PAOX ENSG00000148832.14 0.34

DHRS7 ENSG00000100612.13 0.34

UQCC3 ENSG00000204922.4 0.34

AURKAIP1 ENSG00000175756.13 0.34

RP11-91I20.3 ENSG00000228862.3 0.34

RP11-356O9.1 ENSG00000258414.1 0.34

SLC39A7 ENSG00000112473.16 0.33

SYNGR2 ENSG00000108639.7 0.33

ZDHHC12 ENSG00000160446.18 0.33

HIST2H2AA3 ENSG00000203812.2 0.33

PEX10 ENSG00000157911.9 0.33

AC020571.3 ENSG00000229056.2 0.33

TREX1 ENSG00000213689.9 0.33

GLB1L2 ENSG00000149328.14 0.33

KDF1 ENSG00000175707.8 0.33

MESP1 ENSG00000166823.5 0.33

ARFIP2 ENSG00000132254.12 0.33

AP000438.2 ENSG00000257002.1 0.33

CTNNBIP1 ENSG00000178585.14 0.33

PICK1 ENSG00000100151.15 0.33

RP11-1012A1.4 ENSG00000258466.5 0.33

RPL7P16 ENSG00000242899.1 0.33

HOXA11-AS ENSG00000240990.9 0.33

SLC35F2 ENSG00000110660.14 0.33

ARFGAP3 ENSG00000242247.10 0.33

PMEPA1 ENSG00000124225.15 0.33

NSMCE1 ENSG00000169189.16 0.33

TPRA1 ENSG00000163870.14 0.33

CIB1 ENSG00000185043.10 0.33

DERL2 ENSG00000072849.10 0.33

CBR3-AS1 ENSG00000236830.6 0.33

TRAPPC3 ENSG00000054116.11 0.33

RPL23AP61 ENSG00000280012.2 0.33

STX5 ENSG00000162236.11 0.32

PARK7 ENSG00000116288.12 0.32

MINOS1-NBL1 ENSG00000270136.5 0.32

C19orf48 ENSG00000167747.13 0.32

ZNF613 ENSG00000176024.16 0.32

ARSA ENSG00000100299.17 0.32

RNH1 ENSG00000023191.16 0.32

MSMB ENSG00000263639.5 0.32

CPTP ENSG00000224051.6 0.32

TIMM8AP1 ENSG00000231445.1 0.32

TESK2 ENSG00000070759.16 0.32

RP11-115H13.1 ENSG00000273906.1 0.32

WIBG ENSG00000170473.16 0.32

ERGIC1 ENSG00000113719.15 0.32

SZRD1 ENSG00000055070.16 0.32

TMEM222 ENSG00000186501.14 0.32

BRMS1 ENSG00000174744.13 0.32

ATP6V0B ENSG00000117410.13 0.32

PQLC2 ENSG00000040487.12 0.31

RP11-303E16.2 ENSG00000261061.1 0.31

HIST1H2AE ENSG00000277075.1 0.31

MAP3K6 ENSG00000142733.14 0.31

TNKS1BP1 ENSG00000149115.13 0.31

CLSTN1 ENSG00000171603.16 0.31

PSMD13 ENSG00000185627.17 0.31

PPP1R13L ENSG00000104881.14 0.31

U47924.27 ENSG00000257084.1 0.31

HM13 ENSG00000101294.16 0.31

FOLH1 ENSG00000086205.16 0.31

CD47 ENSG00000196776.14 0.31

TMEM234 ENSG00000160055.19 0.31

ZCCHC6 ENSG00000083223.17 0.31

KIAA2013 ENSG00000116685.15 0.31

ZNF593 ENSG00000142684.7 0.31

C1orf116 ENSG00000182795.12 0.31

RP11-12G12.7 ENSG00000274605.1 0.31

TACSTD2 ENSG00000184292.6 0.31

LRRC26 ENSG00000184709.7 0.31

PDE9A ENSG00000160191.17 0.31

PPIB ENSG00000166794.4 0.31

RPN1 ENSG00000163902.11 0.31

CDH3 ENSG00000062038.13 0.31

NEDD4L ENSG00000049759.16 0.31

CANT1 ENSG00000171302.16 0.31

NWD1 ENSG00000188039.13 0.31

ALDH1A3 ENSG00000184254.16 0.31

ABCC4 ENSG00000125257.13 0.31

PPP1CA ENSG00000172531.14 0.31

TRPM8 ENSG00000144481.16 0.31

ZNF350 ENSG00000256683.6 0.31

CAMKK2 ENSG00000110931.18 0.31

ATP2C1 ENSG00000017260.19 0.31

RP11-855O10.2 ENSG00000250230.2 0.31

TYMP ENSG00000025708.12 0.31

HMGN2P46 ENSG00000179362.14 0.31

P4HB ENSG00000185624.14 0.31

REXO2 ENSG00000076043.9 0.31

MUL1 ENSG00000090432.6 0.31

TMEM251 ENSG00000153485.5 0.30

CTD-2311B13.5 ENSG00000275563.1 0.30

STYXL1 ENSG00000127952.16 0.30

DVL1 ENSG00000107404.17 0.30

PUSL1 ENSG00000169972.11 0.30

RP11-452C8.1 ENSG00000248408.1 0.30

MYDGF ENSG00000074842.7 0.30

PART1 ENSG00000152931.7 0.30

IFI35 ENSG00000068079.7 0.30

CWH43 ENSG00000109182.11 0.30

IDH1-AS1 ENSG00000231908.1 0.30

STXBP2 ENSG00000076944.14 0.30

NUDC ENSG00000090273.13 0.30

PAK1IP1 ENSG00000111845.4 0.30
